# Understanding the distributional effects of recurrent floods in the Philippines

**DOI:** 10.1016/j.isci.2024.111733

**Published:** 2025-01-03

**Authors:** Inga J. Sauer, Brian Walsh, Katja Frieler, David N. Bresch, Christian Otto

**Affiliations:** 1 Potsdam Institute for Climate Impact Research, Transformation Pathways, 14473 Potsdam, Germany; 2 World Bank, Climate Change Group, N.W. Washington, DC 20433, USA; 3 ETH Zurich, Institute for Environmental Decisions, 8092 Zurich, Switzerland; 4 MeteoSwiss, Federal Office of Meteorology and Climatology, Zurich Airport, 8058 Zurich, Switzerland

**Keywords:** Earth sciences, Economics, Global change, Social sciences

## Abstract

Successful recovery from extreme weather events is key to avoid long-term poverty implications. Yet, in disaster prone regions, there may not always be enough time to recover between events. There is a common narrative that the resulting incomplete recoveries aggravate adverse impacts, but approaches allowing for a systematic quantitative assessment are missing. Here, we extend an agent-based model to study welfare effects in the Philippines depending on household exposure and income. We find that incomplete recoveries increase cumulative consumption and well-being losses across the study period 2000–2018 by 40%. While low-income households suffer the highest well-being losses, the effect of incomplete recoveries is most relevant for middle-income households. Consequently, losses can be critically underestimated when drawing conclusions about the impacts of recurrent events based on the impacts of individual events. Accounting for incomplete recoveries may help to better prepare for an intensification of extreme events under climate change.

## Highlights

• We present an agent-based model for household recoveries under recurrent shocks
• Incomplete recoveries lead to additional aggregated consumption and well-being losses
• Low-income households experience the largest well-being losses
• Relative losses caused by incomplete recoveries are largest for middle-income households

## Graphical abstract

**Figure:**
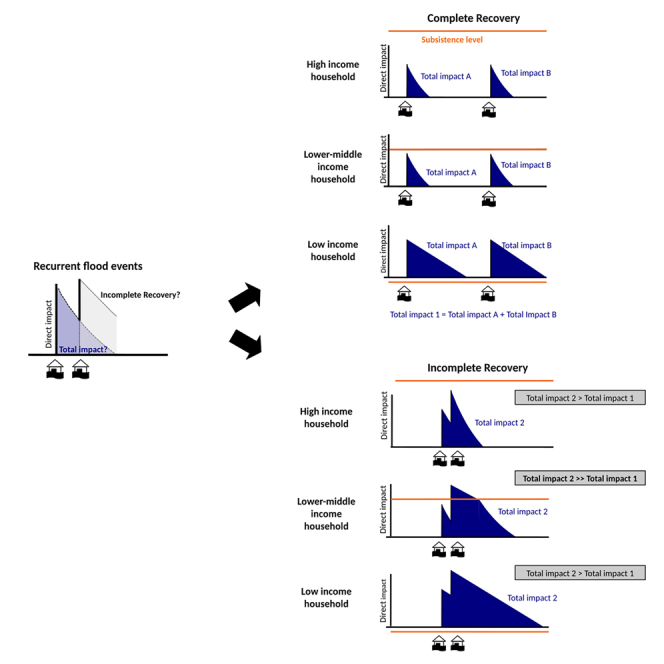

## Introduction

There is increasing empirical evidence that extreme weather events such as floods can have adverse short- and long-term impacts on households’ well-being through persistent losses of income^1^^,^^2^^,^^3^^,^^4^^,^^5^^,^^6^^,^^7^ but also adverse health impacts and food insecurities.^8^^,^^9^^,^^10^^,^^11^ Poor households are more exposed,^12^^,^^13^^,^^14^^,^^15^^,^^16^ more vulnerable,^13^^,^^15^^,^^17^ and take longer to recover than rich households.^18^^,^^19^ In consequence, extreme weather events can have severe poverty implications for poor households,^20^^,^^21^ especially in developing countries with limited means for adaptation and reconstruction efforts in the disaster aftermath.^14^^,^^22^^,^^23^ For instance, it has been shown empirically that households in the Philippines need years to recover from typhoon strikes.^24^ During the recovery phase they accumulate income losses summing up to over 90% of their total economic loss. Further, poor households are disproportionately affected since, in contrast to richer households, they cannot compensate for their income losses in the long run.^24^

Especially in disaster-prone regions households can experience a number of extreme weather events (and associated natural hazards such as landslides after floods) over their lifetime. Depending on a region’s hazard profile, households may be affected by a series of hazards of the same type or by different hazard categories that may overlap spatially and temporally and simultaneously contribute to risk (consecutive events).^25^^,^^26^^,^^27^^,^^28^ One type of independent consecutive disasters are recurrent events, referring to hazards that occur regularly and within short time intervals.^14^^,^^29^ While individual disasters without spatial and temporal overlaps may allow for an unperturbed and full recovery, recurrent events may not allow households to recover completely before the subsequent disaster strikes (incomplete recoveries). When subsequent extreme weather events are spatially or temporally so far apart that they are not linked by any socioeconomic repercussions (e.g., recovery processes), the overall impact of the events can be assumed to be the sum of the impacts caused by each individual disaster (additive impacts). Conversely, when events spatially and temporally overlap they are interconnected through their socioeconomic repercussions and the overall impact is expected to deviate from the mere sum of its parts (non-additive impacts).^30^^,^^31^ However, it depends strongly on the studied systems whether the overall impact is larger or smaller than the sum of the individual impacts.

**Figure 1:**
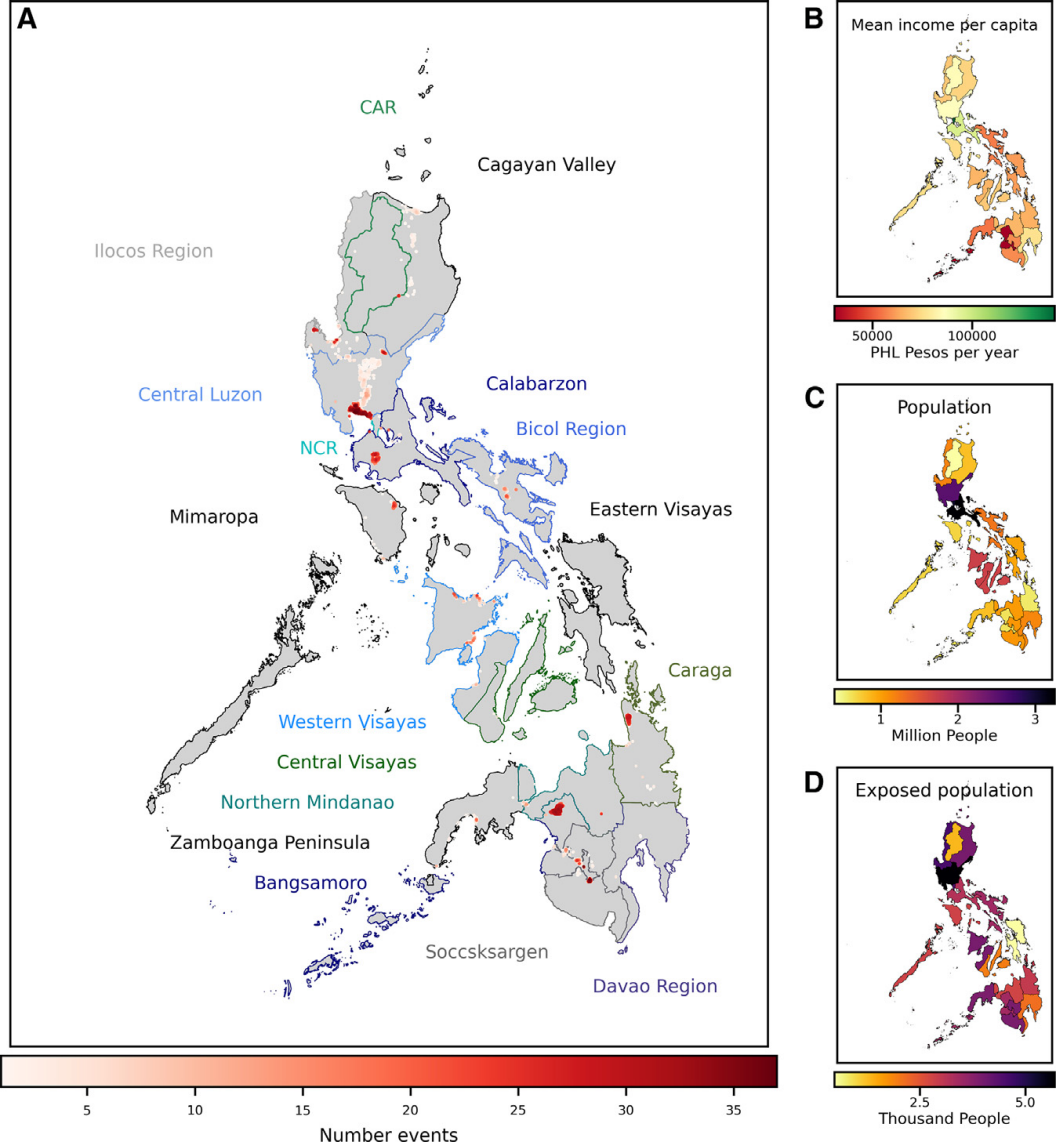
Exposure of the Philippines to floods (A) Regions of the Philippines (colored boundaries) and number events in each grid cell over the period 2000–2018 as reported by the Global Food Database 2021 (red color code). (B) mean per capita income for each region according to FIES 2016 survey,^55^ (C) population numbers, and (D) flood affected people over the period 2000–2018 as estimated from Global Flood Database.^54^

There is a rather extensive body of literature dealing with the spatial overlap of multiple extreme weather events (“multi-layer single hazard” approach).^32^^,^^33^^,^^34^^,^^35^^,^^36^^,^^37^^,^^38^^,^^39^ Furthermore, there have been advances exploring the interactions of different hazards.^40^^,^^41^ However, there has been limited progress on the temporal component and the interaction of impacts from consecutive extremes. Direct impacts can be altered substantially under consecutive climate extremes due to changes in the exposure or vulnerability to subsequent hazards.^42^^,^^43^^,^^44^ For instance, the impact of extreme rainfall and flooding can be altered through a preceding drought,^45^ e.g., due to disadvantageous drainage characteristics of dried out soil. Changes in exposure between events may be caused due to human displacements or asset destruction during a previous event.^42^ In particular, the potential changes in long-term impacts caused by recurrent weather extremes have barely been addressed so far. Furthermore, a comprehensive approach to quantify the distributional effects and poverty implications of incomplete recoveries between recurrent extreme weather events is still missing.^14^^,^^25^^,^^46^^,^^47^ The intensification and frequency increase of extreme weather events under climate change^48^ may render incomplete recoveries more likely in the absence of adequate adaptation and resilience building measures. Therefore, it is important to better understand whether this intensification of extreme events would lead to a disproportionate increase of losses for households to allow for the development of farsighted coping national and local adaptation plans^49^^,^^50^ and climate-proof resilience building strategies.^47^ Even today the Philippines are exposed to multiple natural hazards, including tropical cyclones, floods, earthquakes, and volcanic eruptions.^51^ Over the period 2000–2018 it was ranked as the fourth most climate-affected country in the world, mostly due to the large impacts of tropical cyclones,^52^ but also due to frequent flooding^51^^,^^53^^,^^54^ (Figure 1). Therefore, the Philippines face a large number of natural hazards every year that often occur in a close sequence making it an intriguing case study for the effect of incomplete recoveries.

Agent-based models appear to be well suited to assess the impacts of recurrent interconnected extreme weather events on households because they allow modeling differences in the exposure of households to these events with high granularity. Further, they can account for the heterogeneity of households with regard to their vulnerability and their means to recover from extremes and they allow us to explicitly model the interactions among households in the disaster aftermath.^56^^,^^57^ Thus, differences in the speed and quality of the recovery between various groups of households differing e.g., by their income, social networks, access to financial support, and working opportunities etc., can be modeled explicitly.^20^^,^^58^ This in turn allows us to assess and compare the efficacy and limits of different post-disaster support and adaptation measures.^59^

**Figure 2:**
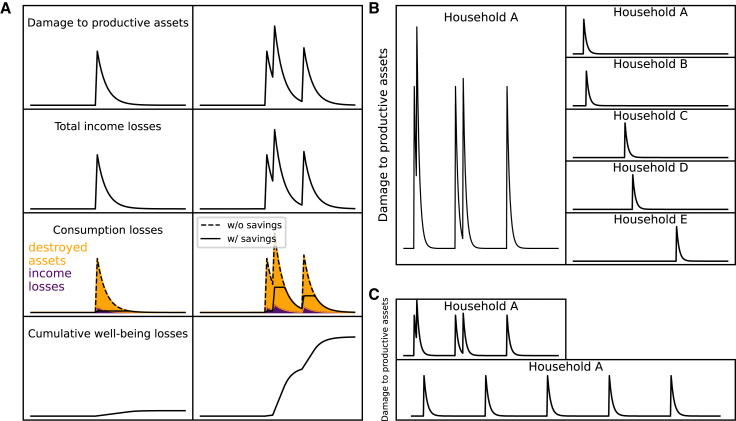
Modeled recovery dynamics for an individual household (A) Recovery dynamics in the aftermath of an individual event (left column) and multiple events (right column). Damage to the stock of productive assets, losses in total income (sum of income generated from assets and social transfers), consumption losses, and cumulative well-being losses (top to bottom). Consumption losses are driven by income losses (purple area) and reconstruction investments to rebuild destroyed assets (yellow area). Dashed and full lines indicate recovery dynamics without and with the opportunity of smoothing consumption losses with savings, respectively. (B) Comparison between the recovery dynamics of the productive asset stock for the factual scenario where households can be affected multiple times (left column) and the counterfactual scenario 1 where shocks are distributed across households such that each household is affected at most once (right column). (C) Comparison between the factual scenario (top) and the counterfactual scenario 2 where the time between shocks is artificially extended so that affected households can always fully recover their assets between shocks (bottom).

In this work, we extend an agent-based model for the recovery dynamics of households in the aftermath of individual disasters^20^ to account for recurrent events. In each region, the model accounts for different groups of households, described by the Family Income and Expenditure Survey (FIES),^60^ which are distinguished by their income and the vulnerability of their physical assets (e.g., quality of housing) to floods (Table S1). We assume that households pay income taxes to the government which redistributes them through social transfers (given in the FIES), independently of flood exposure. Thus, in our model approach the total household income is the sum of their income from productive assets and social transfers (e.g., pensions). The share of income from social transfers is according to the FIES slightly higher for low-income households,^55^ while the absolute amount of transfers increases with the income level Figures S1 and S2B). Flood shocks destroy the productive assets of the affected households (STAR Methods), reducing the households’ ability to generate income to consume and invest into reconstruction of their assets. Households can also be affected indirectly by floods, as social transfers distributed by the government are reduced proportionally to its reduction in income tax revenues in the disaster aftermath (STAR Methods). We assume that in the aftermath of each flood shock, the affected households decide how to distribute their income among savings, consumption, and recovery of their productive assets by intertemporally maximizing their expected well-being over their foresight horizon of 15 years (STAR Methods, Figure 2A). Thereby, they try to keep up consumption for all members of the household above the subsistence line. The subsistence line denotes the consumption level at which households can satisfy their minimum basic needs.^61^ Households can also exist below the subsistence line, but well-being is assumed to be disproportionately diminished. In this work, it is fixed throughout the simulations to the international subsistence level of 350 USD and encompasses basic needs in a narrow sense (e.g., needs of food, shelter, clothing, and medicine^61^). We assume that every household keeps one year’s surplus (difference between income and expenditure derived from the FIES) as precautionary savings. The savings can be used to smooth consumption losses in the aftermath of the disaster (STAR Methods). After completing their recoveries households are assumed to regrow their savings stock linearly within one year, but do not accumulate further savings. We further assume that households cannot foresee future flood shocks or disaster-induced changes in social transfers and therefore do not account for them in the maximization of future well-being (STAR Methods).

Asset damages and consumption losses are common disaster impact metrics. They have the advantage to be measurable in monetary terms and are therefore of great relevance for disaster insurance and fiscal stability measures in the disaster aftermath, respectively.^13^^,^^62^ However, these metrics cannot adequately describe welfare and poverty impacts of disasters for households.^62^^,^^63^ A marginal damage to households’ productive assets can lead to income losses forcing households to reduce consumption. Even if these consumption losses are small, they can be sufficient to push already poor households below the subsistence line where they struggle to satisfy their basic needs. Thus, a marginal loss in productive assets and consumption can result in a large reduction in households’ quality of life and well-being. In our modeling, this is captured by describing well-being as a concave function of consumption such that the same absolute consumption losses cause much larger well-being losses for poor than for rich households (STAR Methods). Further, we use the recovery times of households as a metric to measure the poverty implications of recurrent extremes. We define the recovery time of a household as the time between the shock and the moment when the household has rebuilt at least 95% of its assets. Once vulnerable low income households are pushed below the subsistence line, their recovery becomes very difficult and slow and, in consequence, recurrent events can trap them in poverty.^64^^,^^65^^,^^66^ The slower the recovery of a household, the larger are the (monetized) well-being losses compared to the asset losses. This motivates the definition of socioeconomic resilience as the ratio of asset losses to (monetized) cumulative well-being losses to measure the capacity of households to cope with and recover from disasters. Here, we find that the long-term impacts of recurrent events are larger than the sum of impacts from each individual event, as national cumulative consumption and well-being losses are substantially increased through incomplete recovery between events. While low income households are most vulnerable to floods and suffer the highest well-being losses due to their comparably long recovery times, lower-middle income households perceive the largest additional relative well-being and consumption losses and drop in socioeconomic resilience due to incomplete recovery between recurrent events.

## Results

In a factual scenario, we force the model by spatially explicit exposure to the reported time series of flood shocks (STAR Methods). To this end, satellite-derived flood maps^67^ are combined with population maps^54^ where the estimated number of exposed people is rescaled to match the overall number of people exposed per event as reported by EM-DAT disaster database^53^ (Table S2). As the survey data are not suitable to identify flood-affected households, because the shock-affected people are randomly attributed to households living in affected grid-cells (STAR Methods). Thereby, we ensure that on the regional level the household characteristics of the FIES (e.g., average household size, savings and household income per income group and region) are matched. We additionally distinguish a savings- and a no-savings scenario: in the savings scenario households can smooth consumption losses by their savings, while in the no-savings scenario households cannot employ their savings.

### National-level impacts of recurrent floods

**Figure 3:**
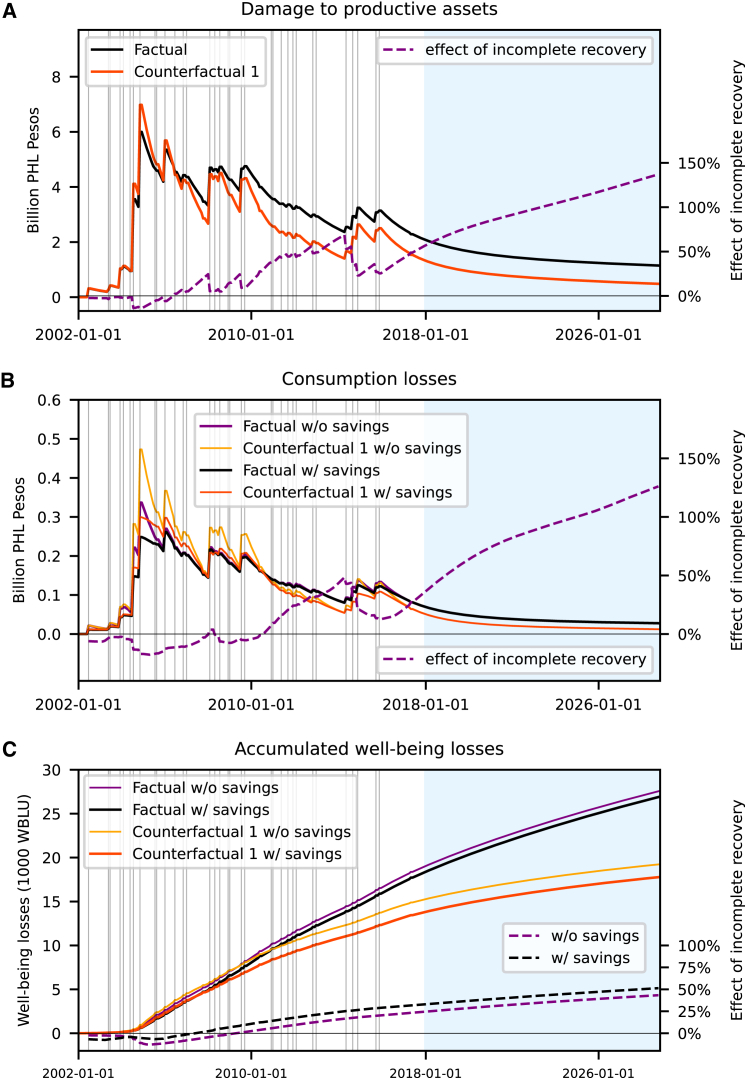
Nationally aggregated response dynamics to observed flood sequence (A) Temporary evolution of the damage to the stock of productive assets, (B) consumption losses, and (C) accumulated well-being losses aggregated over all households to the national level for subsequent flood shock over the period 2000–2018 covered by the Global Flood Database.^54^ The black and red line indicate the factual scenario where the same households can be affected by several floods and a counterfactual scenario where households are affected only once, respectively. Gray vertical lines indicate flood events as recorded in the Global Flood Database from January 1, 2002 to January 1, 2018 (white background). The recovery phase where no further shocks are recorded as the time period is no longer covered by the Global Flood Database is indicated in blue. Dash-dotted lines in (indicate the relative difference between the well-being losses for the factual and the respective counterfactual scenarios. Yellow and purple lines in (B) and (C) indicate scenarios where households do not have savings to mitigate consumption losses. Well-being losses are measured in well-being loss units (WBLU) (Calculation of well-being losses).

The Philippines are affected by floods in nearly every year of the study period 2000–2018. It is even likely that certain households experience even several flood shocks within one TC-prone rainy season in the summer months (Figure 1A). This is why usually not all households affected by a flood event can recover before the next flood strikes (Figure 3). In consequence, the aggregated damages to the households’ assets accumulates also on a national level over time leading to a simulated residual damage of about PHL 2 billion (or 0.04% of the households’ assets) at the end of the shock series. The sequence of events causes residual damages to the national stock of productive assets which takes decades to rebuild even in the absence of further shocks (blue shaded areas in Figure 3). To quantify the effects of incomplete recovery, we compare the factual scenario where the same households can be affected multiple times, with two counterfactual scenarios (CFs): In the first CF scenario, the timing of the floods as well as the number of people affected by each flood remains unchanged but subsequent floods in the same area hit different households so that each household is affected at most once (CF 1, STAR Methods, Figure 2B). In the second CF scenario, floods affect exactly the same households as in the factual scenario but the time span between subsequent shocks has been extended such that there is always enough time for households to recover their assets (CF 2, Figure 2C). The preservation of the timing of the observed shocks in CF1 allows us to single out the importance of households being hit more than once within the study period for the overall impact of incomplete recovery on the national level; whereas, CF 2 allows us to test for nonlinearities in the households’ response as a function of the number of consecutive shocks a household perceives.

Compared to the CF1 scenario, we find a stronger accumulation of the damage to the stock of productive assets over time in the factual scenario (cf. increasing spread between black and orange lines in Figure 3A) resulting in an increase of residual damage by around 50% in 2018 and up to 100% a decade after the last event. The reason is that recovery times increase disproportionately when households fall below the subsistence line which becomes more likely when they perceive multiple shocks (Figure S3). The slightly higher damage to the stock of productive assets observed for the first shocks in the CF1 can also be explained by the incomplete recovery of multiple flood affected households between events reducing the assets that can be destroyed by the floods in the factual scenario. However, after a few shocks this effect is overcompensated by the relatively slower recovery of households affected multiple times.

The damage to the stock of productive assets directly reduces the income of households and thus their ability to consume. This explains why consumption losses follow the dynamics of productive asset damages (Figure 3B). Consumption losses are significantly higher than income losses as households not only have to reduce consumption due to their income losses but also at the expense of reconstruction investments in the disaster aftermath (Figures 3B and S2A). On a national level, the modeled reduction in the income of households that are only indirectly affected is comparatively low due to the relatively small number of directly flood affected households (Figure S2A). Further, incomplete recovery of multiply affected households increases residual consumption losses and well-being losses by around 40% in 2018 and around 100% and 50% after another decade without further shocks, respectively (Figures 2B and 2C). Household savings moderately mitigate well-being losses for the first shocks of the sequence, but do not recover throughout the shock sequence (on a national level). This is why, in the factual scenario, the mitigating effect of savings on cumulative well-being losses saturates toward the end of the shock sequence. By contrast, in the CF1 scenario, household savings mitigate well-being losses throughout the flood sequence since each flood affects previously unaffected households.

### Distributional effects of recurrent floods

**Figure 4:**
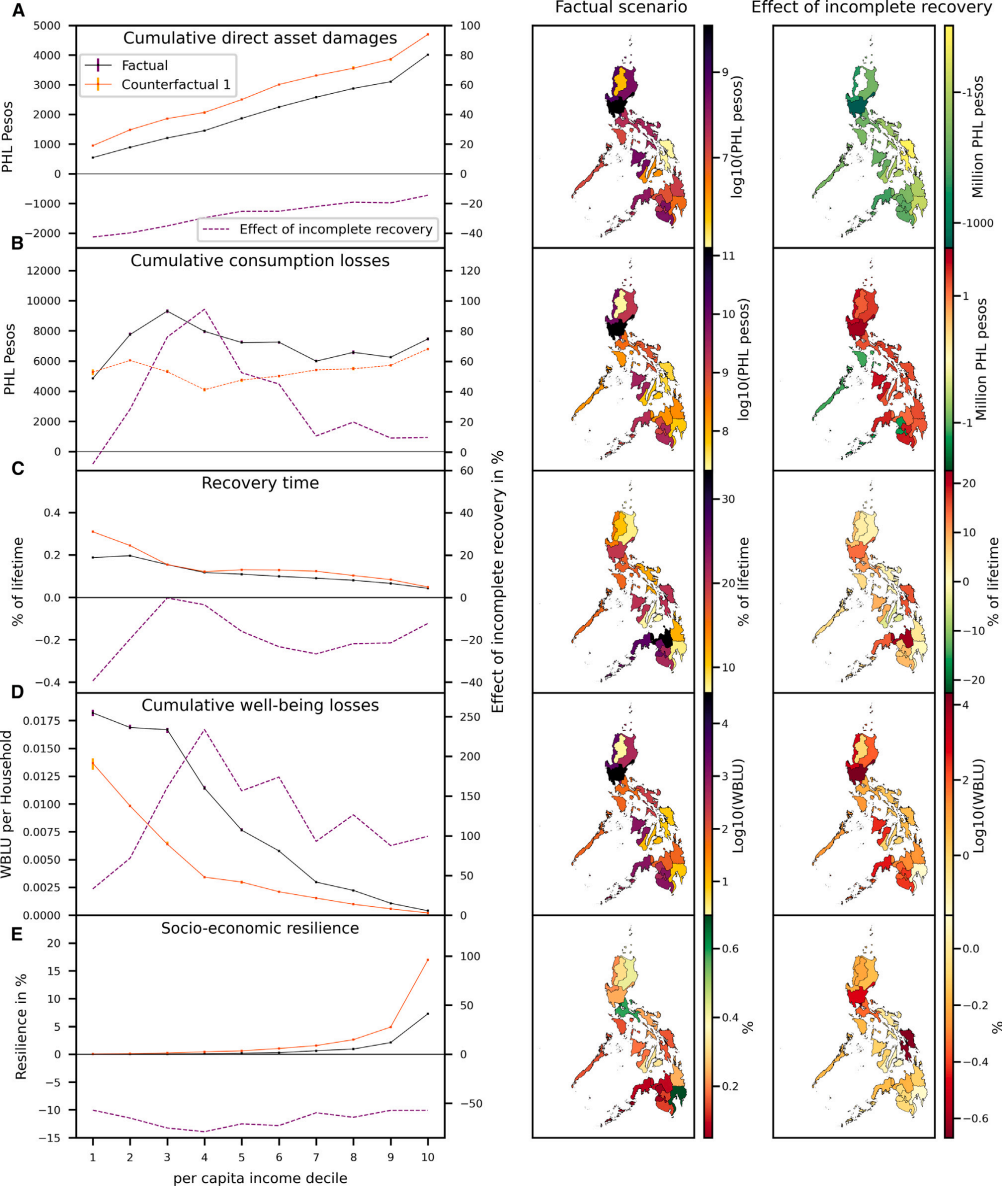
Distributional and regional impacts of recurrent flood shocks (A–E) (A) Average cumulative direct asset damages, (B) average cumulative consumption losses, (C) average share of lifetime spend on recovering damaged assets, (D) average cumulative well-being losses measured in well-being loss units (WBLU) and (E) average socioeconomic resilience. Left column: National averages for each per-capita income decile of the population for the factual scenario where the same household can be affected multiple times (solid black lines) and the counterfactual scenario 1 where each household can be affected at most once (solid orange lines). Absolute (left y axes) and relative differences (right y axes) between the factual and the counterfactual scenarios as they arise from incomplete recovery between events in the factual scenario are denoted by dashed purple lines. Whiskers denote 90% uncertainty intervals as established from 6 runs with varying household selections (Model calibration). Middle column: Regionally differentiated variable values as obtained for factual scenario (color code). Right column: Same as middle column but for differences between factual and counterfactual scenario 1.

The direct asset damages a household perceives from a flood shock depends upon the vulnerability of its assets which are derived from the building structure of its physical assets. According to the FIES survey (Table S2), building standards are positively correlated with household income. Thus, low income households are on average more vulnerable (Figure S4). However, the relatively lower vulnerability of wealthier households’ assets is outweighed by the increase of (physical) assets with income (Figure S6), resulting in an almost linear increase of absolute direct asset damages with income in both, the factual and the CF1 scenario (Figure 4A). They are somewhat lower in the factual scenario because households are not always able to recover assets between shocks. There are three factors determining the recovery speed of a household: (1) the relative damage depending on the asset vulnerability, (2) available construction investments, and (3) social transfers. Since social transfers are only indirectly affected by floods through reduced government tax revenues in the disaster aftermath, they are comparably stable and act as a social security mechanism for directly affected households that lost parts of their productive assets. Thus, households generating a higher share of income from social transfers recover more quickly. Since the share of income generated from social transfers declines only moderately with income (Figure S2B), but asset vulnerability decreases (Figure S4) and available construction investments increase disproportionately with household income (Figure 4A), the time required for asset reconstruction (recovery time) declines with income (Figure 4C). However, asset vulnerability becomes a less important determinant for recovery times under incomplete recovery (Figure S5). On a regional level, the distribution of asset losses largely follows the pattern of exposed population, and the differences between the scenarios are most pronounced in regions experiencing many floods.

The dependence of well-being and consumption losses upon income is more complex than for direct asset damages due to the interplay of two counteracting drivers: On the one hand, absolute direct asset damages increase with income since richer parts of the population have more valuable assets to lose (Figure S6). This requires higher investments in reconstruction resulting in higher consumption losses. On the other hand, richer households can afford to recover more quickly in the disaster aftermath which decreases cumulative consumption losses. Which of these two drivers dominates varies with income level leading to a non-monotonic dependence of consumption losses upon income. For the factual scenario, consumption losses first increase with income for the first three deciles, then decrease for deciles 4–7 due to faster recovery while consumption losses somewhat increase again for the richest deciles (Figure 4B). By contrast, well-being losses monotonically decrease with income across all income deciles, but relatively higher consumption losses result in a slower decay for the first 3 than for the other deciles (Figure 4D). These differences between well-being and consumption losses arise because even comparably small consumption losses can push poorer parts of the population below the subsistence line leading to high well-being losses. Comparing the factual scenario to the CF1 scenario reveals that incomplete recovery increases well-being and consumption losses across all income deciles, except for the poorest decile for which consumption losses are somewhat larger in the counterfactual scenario (Figures 4B and 4D).

Importantly, the effect of incomplete recovery is greatest for middle income households. The reason is that these households recover from individual shocks nearly as quickly as rich households (see weak dependence of the recovery time upon income for deciles 4 to 10 in the CF1 scenario in Figure 4C. However, hit by multiple subsequent shocks they are more likely to come close to — or even fall below — the subsistence line than their richer counterpart. They then have to prioritize consumption over reconstruction efforts which increases their recovery time disproportionately (Figure 4C). By contrast, households in the poorest three income deciles are often pushed under the subsistence line even by a single shock, which critically slows down their recovery.

The regional pattern of well-being and consumption losses are largely determined by population exposure, with the highest losses arising in Central Luzon. We find the largest effect of incomplete recovery on well-being and consumption for middle income regions such as Ilocos Region, Western Visayas, Cagayan Valley and Soccsksargen, but also on Central Luzon. In the poorest region, Bangsamoro, consumption losses even decrease in the factual scenario compared to the CF1 scenario indicating that in this region recovery is relatively slow, and consumption levels are low. After being hit once, the slow recovery of the affected households leads to high and persistent consumption losses. In consequence, additional asset damages from shocks only causes a comparably small additional consumption loss. This is why, in Bangsamoro, overall consumption losses are higher in the CF 1 scenario where more households are hit once compared to the factual scenario where fewer households are hit several times. For well-being losses the effect is reversed. Here, the additional losses of fewer but multiply shocked households outweigh the losses arising when more households are shocked once.

Since rich households can recover much faster than their poor counterparts, the decrease in well-being losses with rising income overcompensates the moderate increase of absolute asset damages resulting in a disproportional increase of socioeconomic resilience with income (Figure 4E). This steep increase of resilience with income is also reflected in the regional resilience patterns. Flood prone but wealthy regions close to the capital region have a substantially higher resilience than less flood-affected but poorer regions in the south of the Philippines (Figure 4E middle column). However, the absolute reduction in resilience due to incomplete recovery is highest for wealthy, flood-prone regions. A comparison of the factual with the CF1 scenario reveals that the socioeconomic resilience is reduced by about 50% when households are not able to recover in between shocks, with a somewhat stronger decrease for lower-middle income groups (Figure 4E right column).

### Frequently hit households suffer disproportional increase in well-being and resilience losses

**Figure 5:**
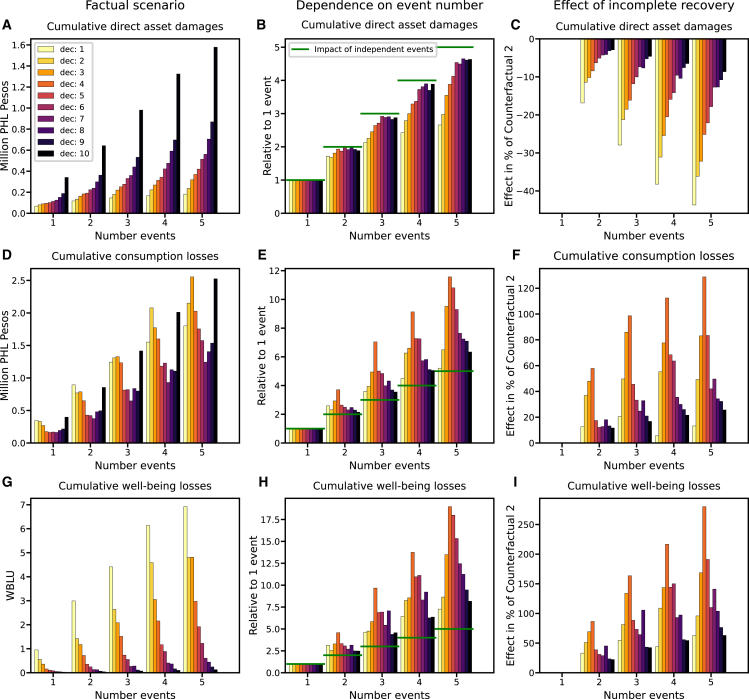
Distributional effects for households in dependence of the number of floods they experience (A) Average cumulative direct asset damages, (D) average cumulative consumption losses, and (G) average cumulative well-being losses for households in each income decile that are affected by 1–5 flood events in the factual scenario. (B, E, and H): Average increase in losses with the number of flood events that households experience relative to the average losses of households that are affected only by one flood event. Horizontal green lines indicate damages and losses that would occur if losses increased linearly with event number. (C, F, and I): Relative increase in average losses in the factual scenario where households may not recover between events compared to the counterfactual scenario 2 where full recovery is always possible. Absolute well-being losses are measured in well-being loss units (WBLU) (Calculation of well-being losses).

We next compare the factual with the CF2 scenario to assess the dependence of the household level impacts upon the number of flood events (Figure 5). First, we note that the direct asset damages a household of the highest income decile suffers from a single flood is higher than the total damages a household of the lowest income decile suffers from five consecutive floods. This disproportionality in losses mainly results from the unequal distribution of assets across income groups (Figure S5) that is not compensated by the reduction of vulnerability with income (Figure S4). Second, across all income groups, direct asset damages increase with the number of flood events (Figure 5A) but increases in damages with event number strongly depend upon the income of the affected households. The reason is that richest deciles can mostly recover in between events as shown by small differences between the factual scenario and CF 2. This results in a nearly linear increase of asset damages with event number (cf. bar height with horizontal green lines in Figure 5B). By contrast, the poorer the household the smaller is its ability to recover in between events, which results in a sub-linear increase in absolute asset damages with event number.

In contrast to asset damage, consumption losses super-linearly increase with event number. In absolute terms, low-income households suffer the largest losses if affected by one or two flood events whereas the loss maximum shifts toward lower-middle income groups for higher event numbers (Figure 5D). The underlying reason is that the recovery of the poorest households living close to — or even below — the subsistence line is slow irrespective of the number of floods affecting them. By contrast, lower-middle income households have sufficient means to recover relatively quickly from one or two flood events but are pushed toward the slow recovery regime in the vicinity of the subsistence line if affected by more than two floods. Upper-middle and high-income groups usually retain enough means for a comparably swift recovery irrespective of the number of floods affecting them. Thus, in relative terms lower-middle income households suffer the largest consumption losses due to incomplete recovery if affected by more than two floods and this relative loss increase becomes more pronounced with event number (Figure 5E). For instance, for households of decile 4, the consumption losses of three consecutive events are larger than of seven independent events (Figure 5E). The comparably high consumption losses of the wealthiest decile of households with regard to households in deciles 5–9 (upper-middle to wealthy) across all event numbers reflects the disproportional high consumption share of the former (Figure S1).

The dependence of well-being losses upon event number is qualitatively similar to the dependence of consumption losses (Figure 5G). Notable differences are that absolute well-being losses are always largest for the poorest decile while, for the richest decile, they remain comparably small irrespective of the event number reflecting the concave dependence of well-being upon consumption. Quantitatively, distributional effects are more pronounced for well-being than for consumption losses. For instance, households of the poorest three deciles experience higher well-being losses from a single flood event than the richest three deciles from five consecutive events (Figure 5G). Similarly to consumption losses, the effect of incomplete recovery is strongest for lower middle income households. For instance, if affected by five consecutive events, the well-being losses for households of these income groups are higher than the losses they would suffer from 18 independent events (Figure 5H).

## Discussion

There is growing concern that the increasing number of extreme events expected under climate change may push people into poverty traps and that recurrent extreme weather events must no longer be considered as a sequence of rare, independent events^14^^,^^25^ ignoring overlapping recovery dynamics. While there is currently only anecdotal evidence for the detrimental effects of incomplete recovery,^14^^,^^25^ we introduce a model and simulation set-up to formalize the testing of this hypothesis and to quantify the effect of incomplete recovery in households between recurrent flood shocks. To this end, we combine a newly available dataset based on satellite imagery for flood shocks^54^ in the Philippines with an agent-based model for the recovery dynamics of households that we extend to account for potential non-additive effects in the households’ response dynamics to recurrent floods.

This approach allows us to go beyond asset damages, the conventional metric of disaster impacts, to assess consumption and welfare losses. This is important for two reasons: first, the extension allows for risk managers to assess and manage disparate welfare costs of extreme weather events, especially to the benefit of low-income households.^12^^,^^14^^,^^17^ We find that discounted consumption losses accumulate over the simulation period to more than twice the cumulative asset damages (+210%). These findings are well in line with purely empirical works finding that most losses of typhoons to households in the Philippines arise from income losses in the disaster aftermath and not from the direct impact of the storms on households’ assets through strong winds, rainfall and storm surge.^24^ Further, the poorest are most vulnerable to floods and suffer the highest well-being losses among all income groups. Living already close to the subsistence line, they struggle to fulfill their basic needs such as sufficient access to clean water, education, and healthcare even in times without disasters. This leaves them with little means to recover from flood shocks^68^ resulting in a slow recovery and an elevated risk of being trapped in poverty^17^^,^^64^ by subsequent flood events. We find incomplete recovery to substantially increase cumulative consumption and well-being losses by 40% (Figure 3C), respectively, over the study period. Importantly, lower-middle income households perceive the largest additional relative well-being losses (+280%) and (Figure 5I) consumption losses (+130%) (Figure 5F) and drops in socioeconomic resilience due to incomplete recovery in the historical period. While these households have enough financial means to recover relatively quickly from an individual flood, recurrent floods can push them close to — or even below — the subsistence line, critically slowing down their recovery and driving up well-being and consumption losses. There are studies that support our findings, highlighting the difficulties for recovery in a multi-hazard environment, where recurring extreme events undermine a recovery to the pre-disaster state.^69^^,^^70^ In contrast, recurrent extremes may also act as a trigger for adaptation and coping strategies. An empirical study on the impacts of Tropical Cyclones on households in the Philippines shows that private and public cash transfers currently help to moderate the adverse distributional impacts of recurrent tropical cyclones.^71^ This highlights the need to take into account ongoing coping and adaptation efforts, to assess their effectiveness also under increased hazard frequencies and intensities caused by climate change.

Our modeling approach highlights that many important dynamics are missed when floods are considered as rare, independent events which without adaptation and coping strategies lead to a critical underestimation of losses and a non-optimal allocation of reconstruction and resilience-building efforts that do not appropriately account for the differential needs of the various income groups of households. Regarding short-term interventions and support, our study highlights that in order to reduce the critical well-being losses of the poorest, it is not effective to reduce consumption losses uniformly across all income groups. Instead, consumption losses should be reduced with a focus on efficient well-being improvements.^15^ The finding that recurrent floods can push even middle income households below the subsistence line suggests that support measures should be tailored toward the post-disaster needs of these groups (in addition to the poorest part of the population). Putting these insights together, welfare and consumption-informed risk management strategies awareness leads to more and cheaper opportunities for risk mitigation and recovery. More opportunities, because we are able to assess the welfare and economic benefits of investments and interventions that do not affect asset damages. Cheaper opportunities, because distributional awareness informs more targeted and efficient investments.

As the model comprises several parameters estimated from various sources (Table 1) such as the subsistence line, the basic recovery rate of households below the subsistence line, the productivity of capital stock, and the planning horizon, we test the sensitivity of the main statements on the variation of these parameters. We find that the major outcomes of our study are not sensitive to moderate changes in these parameters (Figures S7–S17). Additionally, the sensitivity assessment allows us to compare the effects of variations in these parameters on welfare metrics nationally and across different income deciles. Overall consumption and well-being losses and distributional impacts are particularly sensitive to the minimum recovery rate and the subsistence line (Figures S9, S10, S17, and S18), while the productivity of capital mainly affects overall losses and is less relevant for the distribution of losses. We identify two parameters that can be directly linked to coping opportunities: (1) an increase in the minimum recovery rate can be understood as support for people living below the subsistence line to reconstruct, which would moderate the consumption and well-being losses of the low and lower-middle income households; and (2) the subsistence line cannot be directly shifted by policies, but one could understand the direct provision of basic goods in the aftermaths of a disaster as a lowering of the subsistence line. Both measures would be directed coping measures, targeting affected low- and middle-income households. The sensitivity to both parameters indicates that there may be substantial benefits of directed reconstruction support. Total well-being losses are particularly sensitive to the productivity of capital stock (Figure S8), but do not effectively mitigate distributional impacts, as higher productivity of capital stock decreases absolute consumption losses across all income groups almost equally, but slightly stronger for high income groups (Figures S12 and S16). Although, well-being of high income households is still less sensitive to changes in this parameter, increases in the productivity of capital stock do not efficiently smooth distributional effects (Figure S16). Additionally, this parameter cannot be influenced by politics, but our analysis suggests that households with a strong reliance on physical capital are more vulnerable to shocks than those supported by social transfers (Figure S5). Our analysis shows that results across all metrics are rather insensitive to the planning horizon (Figures S7, S11, and S15). This is rooted in the assumption that households cannot foresee any future shocks. If they perceive a second shock within their reconstruction phase, they need to adjust their recovery plans.

**Table 1:** Summary of all calibrated parameters

Parameter	Abbreviation	Value	Source	Unit/Format
Event dates	–	–	GFD (Table S2)	yyyy-mm-dd
Affected people of each event	–	–	EM-DAT (Table S2)	people
Household income	i_h_∗	–	FIES	PHL Pesos/year
Consumption at the subsistence line	c_sub_	15925 [11735; 18200]	FIES	PHL Pesos/year
Recovery spending of people living under the subsistence line	R_sub_	3339 [1486; 4825]	FIES	PHL Pesos/year
Precautionary savings	s_h_∗	–	FIES	PHL Pesos
Household vulnerability	ν_h_	Derived from FIES building standards: robust = 0.14 ± 0.06 moderate = 0.4 ± 0.08 fragile = 0.7 ± 0.14	FIES and ref.^20^	–
Planning horizon	T_p_	15 [10; 20]	Extended from ref.^20^	years
Total runtime	T_sim_	162	This paper	years
Productivity of capital	π	0.33 [0.2; 0.45]	Ref.^20^	–
Elasticity of the marginal utility	η	1.5	Ref.^20^	–
Future discount rate	σ	0.06	Ref.^20^	1/year

In this work, we focus on the contribution of incomplete recoveries to the distributional and poverty effects of recurrent floods in the Philippines. However, since the channels through which extreme events impact households may be similar for other major event categories, our findings are likely transferable to other countries affected by extremes of different or multiple categories (multi-hazards). Importantly, our results underline the inadequacy of development policies targeted on “easy wins” to reach adaptation and development goals, for instance, by lifting poor households just above the poverty line.^21^ Being not always able to recover between floods, these households could be pushed again below the poverty line by recurrent floods. Thus, we may conclude that to reduce chronic poverty, adaptation strategies that foster the recovery speed of households are needed. Our study claims for an extension of social protection programs from the poorest to larger parts of the society in order to allow for transformative processes required for climate change adaptation.^72^ Further, our insights on additional well-being losses due to incomplete recoveries could be helpful for countries where interconnected extreme weather events have played a minor role in the past but may become important with the intensification of these events under global warming. The costs of inaction or even maladaptation^73^ could be especially high in these countries since the intensification of extreme weather events may aggravate the problem of incomplete recovery, especially for low and lower-middle income populations in the absence of adequate resilience building efforts. Our study may therefore encourage the incorporation of impact interactions within multi-hazard environments in National Adaptation Plans,^74^ as well as in national and international disaster risk reduction strategies.^75^^,^^76^

### Limitations of the study

The households’ resilience model comprises several simplifying assumptions and parameter uncertainties (Table 1). First, to keep the household response to the shock tractable despite the large number of households and shocks, we have made several simplifying assumptions regarding the disaster response, and the response options of households. In our model, flood affected households decide upon their reconstruction investments – and thus their recovery speed – by minimizing their expected well-being losses in the recovery phase. They thereby do not factor in the risk that another shock hits before they have recovered. This simplifying assumption is motivated by empirical findings from the behavioral economics literature showing that households have difficulties to appropriately account for tail-risks such as the risk of rare extreme events even if these can have potentially catastrophic outcomes.^77^^,^^78^ Reasons for the low perception of risk is that households are often not fully aware of, or are not well-informed about, the risk.^79^^,^^80^^,^^81^ Importantly, our modeling accounts for the possibility that households are risk averse and accumulate precautionary savings in anticipation of financial shocks resulting from e.g., economic downturns, family tragedies, but also disasters such as extreme weather events. The level of savings that households consider to be the optimal tradeoff between a higher consumption in normal times and a faster recovery in the aftermath of a shock is directly derived from the FIES survey data. This allows us to account for the heterogeneity among households with regard to their willingness and ability to accumulate savings. However, it may happen that at the time of the survey households exceed or do not maintain their optimal savings level; for instance, because they just experienced a shock and we then nevertheless consider their current level of savings as optimal. Further, from a modeling perspective, this has however the drawback that this level cannot be determined endogenously from the decision rationales of the household agents. In this case, we nevertheless consider their actual level of savings as optimal. Further, to keep the households’ problem simple, we make simplifying assumptions on how households recover their assets (STAR Methods). For instance, in the common case that households are not pushed under the subsistence line by the flood shock, we assume a generic exponential recovery with a recovery rate determined through the household’s minimization of its expected well-being loss. However, we would not expect that our main findings are sensitive to these simplifying assumptions.

Further, in the model, the households do not anticipate any changes in government support which could increase when the government takes up debt to support affected households or decrease due to dwindling tax revenues limiting the government’s ability to support households through social transfers. Further, households do not factor potential surges in international aid in the disaster aftermath into their recovery decisions. As the Philippines are a major recipient of international grants on disaster risk reduction and management,^82^ these are definitively simplifications that may nevertheless be reasonable because individual households may not be able to anticipate the response of the government – which likely depends upon many factors including the fiscal policy of the ruling party – nor the response of the international community – which likely depends upon how severe the flood in the Philippines is considered compared to other ongoing crisis situations. In 2010, the government enacted the Philippine Disaster Risk Reduction and Management Act of 2010 (or Republic Act 10121), as the country’s most important legal instrument and guiding policy framework driving DRRM momentum across various governance levels. Although the Local Disaster Risk Reduction and Management Fund (LDRRMF), amounts to 5% of income from regular sources, of which 30% are dedicated to quick disaster responses, local authorities often face significant challenges in securing adequate resources for post-disaster support, including reconstruction of critical infrastructure, delivery of services, and support for livelihoods.^83^

Aiming to stay as close to the survey data as possible, we account for the heterogeneity with regard to the households’ access to social transfers (which can be derived from the FIES survey data). We do not account for the ability of households to finance reconstruction investments through taking up credits or lending from non-affected households nor do we model differences in the access of households to credits or lendings such as debt-related borrowing constraints income^84^ since these are not included in the FIES data. Further, since we cannot derive data on individual adaptation decisions from the FIES database, we assume that households do not invest in adaptation in the sense that they decrease their vulnerability in the aftermath of flood shocks, i.e., they do not invest in “building back better”.

This data-driven modeling approach is limited in the sense that it may result in an underestimation of the recovery speed and socioeconomic resilience of those households that have access to credits or financial support from their social network, but it allows keeping the model relatively simple and tractable. Further, by comparing households’ well-being losses with and without accounting for household savings, we find that, while our main finding regarding the highest additional well-being losses for middle-income households under recurrent shocks remains robust, the savings damp the well-being losses for the highest income deciles (Figure S18). We would expect that introducing the ability of households to borrow and lend could have a similar effect on well-being losses: low-income households might have comparably fewer options to reduce their well-being losses by borrowing than higher-income households.

Focusing on the recovery dynamics of households, we aim to keep the modeling of the rest of the economy simple by assuming a closed national economy without financial flows from and to the Philippines. This may be a substantial limitation since post-disaster support as international aid and remittances play an important role during the recovery process.^20^^,^^82^^,^^85^^,^^86^ We here decided to nevertheless focus on an “alone-out-in-the-dark” scenario where neither international post-disaster support nor additional adaptation efforts during the study period^87^^,^^88^ are taken into account in order to reduce the number of confounding drivers of the households’ recovery dynamics. This allows us to gain a better understanding of the main mechanisms through which interconnected floods affect the households’ recovery dynamics and thus household consumption and well-being.

Moreover, we do not (1) consider a labor market in the model and (2) explicitly model critical infrastructure and other public physical capital. Both are potentially important channels through which floods impact on households’ recovery^58^ and the economy in general.^89^ Resolving these channels in future versions of the model would also allow assessing adaptation options such as the hardening of critical infrastructure explicitly.^84^ The model set-up provides a promising framework to test the effectiveness of a broad portfolio of adaptation and coping strategies. In particular, the reasonable “need-based” allocation of public spending in the aftermath of disasters may be a key determinant for achieving Sustainable Development Goals and the targets set in the Sendai Framework for Disaster Risk Reduction.^90^ Additionally, the modeling framework could be extended to assess decisions of individual households to reduce their vulnerability to flood shocks by investing in adaptation. For instance, following^91^ households could invest in adaptation when they expect the marginal benefit of an adaptation measure to exceed the sum of its marginal costs and marginal externalities.

Further, we base the modeling on the socio-economic situation of the year 2015 and neglect population growth, within-country migration, and potential income-specific exposure changes over the study period 2000–2018. Therefore, we may overestimate the number of events individual households experience, as some households may move to less exposed areas. In consequence, the effect of incomplete recovery on a national level may be overestimated. On the other hand, the reported number and extensions of floods is likely to be underreported in the GFD database due to missing events in the reporting and cloud cover that may obscure parts of the flooded areas.

## Resource availability

### Lead contact

Requests for further information and resources should be directed to and will be fulfilled by the lead contact, Christian Otto (christian.otto@pik-potsdam.de).

### Materials availability

This study did not generate new materials.

### Data and code availability

• **Data:** The flooded areas used to derive flood affected households in this study are available from the Global Flood Database: https://global-flood-database.cloudtostreet.ai/.^54^ The gridded population data analyzed in this study are available from the Gridded Population of the World database: https://sedac.ciesin.columbia.edu/data/collection/gpw-v4.

The number of affected people used to calibrate the number of affected people are available at the EM-DAT repository: https://www.emdat.be/.^53^

The FIES survey data analyzed during the current study are not publicly available as they need to be requested from the Philippines Statistical Authority, but may be made available by the corresponding author upon reasonable request.

• **Code:** All the source code required to reproduce the results of this work is available at github: https://github.com/ingajsa/hhrm_recurrent_events/tree/v1.1. The results generated during this study and the scripts required to reproduce all figures are available at zenodo: https://zenodo.org/records/14497296.
• **All other requests:** For any further questions that may arise when replicating the analyses of this paper, please address the lead contact.

## Acknowledgments

This research has received funding from the 10.13039/501100002347German Federal Ministry of Education and Research (BMBF) under the research projects PIK CHANGE (01LS2001) and QUIDIC (01LP1907A).

## Author contributions

I.S., B.W., and C.O. designed the method. I.S. conducted the simulations. All authors contributed to the analysis, and I.S. and C.O. wrote the manuscript with contributions from all authors. All authors discussed the results.

## Declaration of interests

The authors declare no competing interest.

## STAR★Methods

### Key resources table

**Table:** 

REAGENT or RESOURCE	SOURCE	IDENTIFIER
**Deposited data**		

Flooded areas	Tellmann et al. 2021	https://global-flood-database.cloudtostreet.ai/
Gridded Population Data	Doxsey-Whitfield et al. 2015^67^	https://earthdata.nasa.gov/data/projects/gpw
Affected People EM-DAT	EM-DAT, CRED	https://www.emdat.be/
Data required to reproduce findings	This paper	https://zenodo.org/records/14497296
Code required to reproduce findings	This paper	https://github.com/ingajsa/hhrm_recurrent_events/tree/v1.1

**Software and algorithms**		

Household resilience model for recurrent events	This paper.	

### Experimental model and study participant details

The study examines groups of househholds that are differentiated by: i) their exposure to floods and ii) socioeconomic characteristics such as income level, sources of income and savings as derived from the FIES database.

### Method details

#### Flood data

To generate a realistic sequence of flood affected areas for the Philippines, we use spatially explicit flood (8” 222m) maps from the Global Flood Database (GFD)^54^ based on satellite imagery. It lists 44 flood events in the time period 2000-2018 (Figure 1) in the Philippines and comprises information on displaced people. In general, the database groups flood events into one of the following categories: i) Heavy rain; ii) Tropical Storm, Surge; iii) Snowmelt, Ice, Rain; iv) Dam. In the Philippines all events are grouped into either category i) or ii). Here, we include all the flood events given in the database regardless of the event type.

#### Socio-economic data

Household characteristics (total income, income from social transfers, building standards, location (regions-ADM1) are extracted from the Family income and Expenditure Survey (FIES).^55^ The FIES provides household census data on income, consumption, and living conditions representative for all households living in the Philippines. As not all households can be sampled, questioned households receive a weight indicating how many households it represents. The survey data is representative on the regional level, so it is not possible to extract the exact coordinates of a household. The national population distribution on a 30” ( 1km) resolution of the Philippines in 2015 is derived from the gridded-population-of-the-world (GPW v4) dataset.^67^ For model calibration we use flood affected people from The International Disaster Database (EM-DAT)^53^ for each event.

#### The agent-based household resilience model

We present an extension of the household resilience model of ref.^20^ that allows simulating the households’ response dynamics to recurrent extreme weather events (cf. Figures 1 and 2). The model describes an economy consisting of heterogeneous households that differ, among other things, by their income, vulnerability, and exposure to extreme weather events, and a government that collects income taxes and distributes the tax revenues as social transfers to the households. The total income of a household $i_{h}\left(t\right)$ at each point in time is composed of income from its stock of productive (physical) assets (capital stock) $i_{h}^{k_{h}^{eff}}=\pi k_{h}^{eff}\left(t\right)$ and its income from social transfers $i_{h}^{sp}\left(t\right)$,

(Equation 1) $$i_{h}\left(t\right)=i_{h}^{sp}\left(t\right)+\left(1-\delta_{sp}^{tax}\right)i_{h}^{k_{h}^{eff}}\text{,}$$

where we have introduced the income tax $\delta_{sp}^{tax}$ and the productivity of capital π. Productive assets are not provided by the FIES. Therefore, we assume that all the income given in the FIES that is not provided by social transfers, is generated from the productive asset stock, which we estimate from the income and the productivity of capital π. Consequently, in this work, we treat income from labor like income from productive assets. In our model setup, we distinguish only between two different types of expenditure: i) expenditure on consumption and ii) expenditure on recovery. During the recovery phase, total expenditure may exceed income as savings are used to compensate consumption losses. After the reconstruction of the capital stock, households save until reaching their steady state level of savings. In this second financial recovery phase, income may therefore exceed expenditures on consumption. The households’ consumption $c_{h}\left(t\right)$ varies with its income $i_{h}\left(t\right)$, disaster response, and precautionary savings $s_{h}\left(t\right)$ as detailed in Secs. Economic equilibrium and Recovery dynamics. The utility a household gains from its consumption is described by a standard constant relative risk aversion (CRRA) utility function

(Equation 2) $$u_{h}\left(t\right)=\frac{c_{h}{\left(t\right)}^{1-\eta }}{1-\eta }\text{,}$$

where the elasticity of the marginal utility of consumption η, expresses that a unit change in utility affects the well-being of poorer households more than of richer households. It represents both the risk aversion and the aversion to inequality in a society and is linked to preferences and values. The households’ cumulative well-being is then the time integral of the utility function over the simulation time $t_{sim}$

(Equation 3) $$W_{h}=\int_{0}^{t_{sim}}u\left(t\right)dt$$

Government income consists of the taxes collected from all households $i_{g}\left(t\right)=\sum_{h=0}^{N}w_{h}i_{h}\left(t\right)\delta_{sp}^{tax}$, where N denotes the number of modeled households and $w_{h}$ is the household weight measuring the part of the Philippines’ population that is represented by the household agent in the model. Government expenditure is given by the sum of the social transfers $i_{h}^{sp}$ disbursed to the households $C_{sp}\left(t\right)=\sum_{h=0}^{N}w_{h}i_{h}^{sp}\left(t\right)$. We assume that the government’s fiscal balance remains always in equilibrium, i.e., at each time-step government income equals government expenditure.

#### Derivation of flood forcing and selection of affected households

To obtain a good representation of the flood shocks with regard to coverage and timing, we make use of flood records from satellite images and event recordings from the EM-DAT database and build shock time series for individual households. Household attributes such as income, location (region) and asset vulnerability are extracted from the FIES such that the model agents represent the entire population of the Philippines. The flood shocks are modeled as specific time stamps at which the households affected by the event are shocked. The agents are modeled over a time period of 16 years where households can experience shocks complemented by an additional period afterwards until all households have fully recovered (162 years) to ensure the comparability of the factual and counterfactual scenarios. Beginning in 2002, flood events take place at the event dates given in Table S2. The time resolution of the model is four weeks, so events that lie within four weeks are modeled as one event. In previous studies on recurrent flooding, the use of self-reported shocks has proven to be very useful for assessing income losses.^1^ However, the FIES does not include an adequate representation of self-reported shocks over the observed time series. Therefore, we apply a three step procedure to generate a historical time series of flood affected households for the factual scenario: we i) intersect the GFD flood maps with the population map^67^ to determine all flood affected grid cells, ii) distribute individual households from survey data to grid cells to simulate the exact location of the households, and iii) derive affected households from the flooded grid-cells to match the number of affected people recorded in EM-DAT.

In step i), we use the open source climate impact modeling tool CLIMADA^92^ to generate maps of exposed population for each flood event. As input data, we use the satellite maps from the Global Flood Database (GFD)^54^ and the population map from 2015 from the gridded-population-of-the-world (GPW v4) dataset.^67^ For step ii), we use the union of flooded populated grid-cells from all events to distribute households included in the FIES^54^^,^^55^ according to the population given in GPW in each populated flooded grid-cell. The FIES does not account for the exact locations of households and can only be assumed to be representative on the regional level.^55^ Therefore, for each region we select all households sampled in this region from the FIES. We cannot make any reasonable assumptions about income differences between single grid cells within one region, so we aim to fill grid cells with heterogeneous households. As sampled households have an assigned weight and represent a group of households of the same type, we “unpack” each group of households, receiving several single households with the same characteristics. We fill each flooded grid cell with single households from different groups, such that the household representation in a grid-cell is as diverse as possible. The remaining households are considered as being located outside the affected areas. They are not shocked, but are also included in the model simulation with their remaining weights as they contribute to the consumer tax revenue of the government. Comparing data records from EM-DAT with the total number of affected people generated by the intersection of the flood map with the population data, we find that usually the number of people in all affected grid cells exceeds the number of affected people recorded in EM-DAT (cf. Table S2). Therefore, we introduce step iii) and calibrate the model for each event to the number of affected people given in EM-DAT (cf. Model calibration). For each event, we calculate the ratio of affected people in the EM-Dat and the exposed population calculated with CLIMADA. We apply the resulting fraction as a probability of being flooded on each grid cell and randomly choose only the fraction of households from all households located in the grid cell (Table S2). To generate one realization of a shock time series, steps i) — iii) are repeated for all events. In total, we generate 6 different shock time series by repeating the household distribution step ii) to investigate the robustness of our work. In order to derive time series of flood affected households for the counterfactual scenario 1, we also undertake step i) and iii) to derive the exact number of affected people in each region. In contrast to the factual simulation, we do not distribute households to grid-cells, but for each region only select households from the survey until the number of affected people is reached. Households that have been affected in a previous event cannot be affected again in a subsequent event. Thereby we make sure that each household is only affected once, but the total number of affected people remains unchanged compared to the factual scenario. In the counterfactual 2 each households experiences the same number of flood events as in the factual scenario, but the time stamps are manipulated such that households always fully recover between events.

#### Basic model assumptions on recovery and their validation

The validation of the overall model performance and the plausibility of key assumptions is limited by four main aspects:

1. Temporal resolution of empirical data: In order to follow recovery tracks of the households, panel data with a high (sub-annual) temporal resolution) would be required. Publicly available survey data such as the FIES database usually have only multi-annual resolution (e.g., 4 year resolution in case of the FIES).^55^^,^^93^
2. Inconsistent and incomplete self-reporting: From the survey data, it is impossible to conclude whether and when individual households were hit by a flood. Therefore, studies based on the FIES typically use modeled hazards and intersect them with households’ locations.^24^^,^^71^ Additionally, only households that, for a given year, participated in both waves of the survey are included in the FIES. One of the reasons for missing the second wave of the survey is the destruction of their housing due to disasters like typhoons, floods or fires.^71^^,^^94^ This may reduce the overall number of flooded households in the sample. This is one of the reasons why, the outcomes between the analyses of self-reported shocks and physical indicators or modeled hazards may differ. Consequently, capturing the history of experienced events of households already presents a major challenge and complicates the tracking of the recovery trajectory of individual households.
3. Closed economy and “Alone-out-in-the-dark scenario”: In this work, we aim to provide results for a scenario without any adaptation, coping and international disaster support. Additionally, empirical data on income or consumption is subject to influences from disaster unrelated drivers, e.g., growth of families, illness, political change, economic crises, or social conflicts. These influences are difficult to account for, especially if we aim to describe the exact recovery pathway. For the Philippines, a recent study strongly suggests that unconditional cash transfers, as well as several advanced adaptation policies and strategies on emergency response and disaster risk reduction, helped to moderate both the overall and the distributional impacts of recurrent tropical cyclones.^71^
4. Inconclusive results from different case studies: Case studies on recovery or long-term impacts using local longitudinal data come to different conclusions depending on the local context, disaster severity and the recovery indicators under consideration. For example, in cases of Myanmar and the US low income areas experience higher relative damage and need more time to recover after floods and tropical cyclones,^95^^,^^96^ while in a case study for Manila it was found that income and houses of low income households recover faster, due to their simpler building standards and the increasing demand on low qualified labor in the aftermath of a disaster.^58^

Nevertheless, the key assumptions on household recovery pathways can be motivated by empirical findings on recovery pathways from other case studies as well as by simple rationales of the household agents:

• Higher relative damage to low income households: In this work, we assume a higher vulnerability of low income households based on the building quality standards given in the FIES (Figure S4; Table S1). On average, we assume a relative damage of around 30% for the lowest income group and 15% for the richest households. Comparing these assumptions to an empirical case study for two floods in Myanmar, shows a stronger difference between the highest incomes and the lowest (low income households: 42.8%–69.4%; high income households: 6.52%–20.8%).^96^ Consequently, for high income groups our initial vulnerability estimates are within the same range, while for low income groups they are lower. However, the relative damage experienced especially by low income households under consecutive events in our study can be much larger than the vulnerability factors given in Figure S4 and Table S1 (Equation 8), as vulnerability factors are applied to the remaining asset stock, which can be much smaller than the original asset stock under slow recovery. Therefore, we consider the vulnerability estimates in reasonable ranges both from qualitative and a quantitative perspective.
• Exponential recovery pathways of households above the subsistence line: Due to the data constraints regarding panel surveys, recovery pathways are mostly tracked on an aggregated level (e.g for entire areas or across different sectors),^97^^,^^98^ applying e.g. remote sensing methods to investigate physical recovery. Observed recovery tracks show both exponential and linear behavior superimposed by strong noise.^95^^,^^98^ Yet, exponential recovery is often a sign of more efficient recovery. In this work, we choose exponential recovery as the first option of recovery, as in terms of smoothing well-being losses, it is the most efficient recovery type and therefore the first choice of our rational agents.
• Constant recovery rate for people living below the subsistence line: There is a wide range of literature that underlines the importance of social vulnerability and the preexisting conditions for impact and recovery outcomes.^99^^,^^100^^,^^101^ As the differences in these preexisting conditions need to be reflected in the model assumptions, we choose a less efficient recovery pathway for people in insecure economic conditions. Additionally, from a rational perspective, people living below the subsistence line have no means to recover at all. However, due to informal support, e.g, through unconditional cash transfers^71^ and their social relationships, they are likely to recover to some degree.
• Households avoid to fall below the subsistence line: As a boundary condition, the model is based on the assumption that households avoid to fall below the subsistence line and recover slower once they reach this critical level. This assumption seems plausible since, to survive, households are forced to fulfill their immediate basic needs and would therefore likely prioritize consumption over investments at these critical levels of income. There is also some empirical evidence that poor people tend to prioritize consumption over investments in productive assets.^102^

### Quantification and statistical analysis

#### Economic equilibrium

In the absence of shocks, i.e., in equilibrium, the economy is in a steady state. In the following, we denote the steady state values of variables by a subscript ${\left(\cdot \right)}^{*}$. The steady state values of total household income $i_{h}^{*}$ and income from social transfers $i_{h}^{sp,*}$ are taken from the FIES survey. The steady state value of the social transfers then reads $C_{sp}^{*}=\sum_{h=0}^{N}w_{h}i_{h}^{sp,*}$. Since the government’s budget is balanced, the income tax revenues must equal the costs from social transfers allowing to derive the income tax rate as,

(Equation 4) $$\delta_{sp}^{tax}=\frac{C_{sp}^{*}}{\sum_{h=0}^{N}w_{h}i_{h}^{k_{h}^{eff,*}}}\text{.}$$

The steady state value of each household’s asset stock can then be estimated from its income $i_{h}^{*}$ as,

(Equation 5) $$k_{h}^{eff,*}=\frac{i_{h}^{*}-i_{h}^{sp,*}}{\left(1-\delta_{sp}^{tax}\right)\pi }\text{.}$$

Further, the steady state value of the national stock of physical assets is given as $K^{*}=\sum_{h=0}^{N_{h}}w_{h}k_{h}^{eff,*}$. In the steady state, households consume all of their income $c_{h}^{*}=i_{h}^{*}$ and keep a constant stock of precautionary savings $s_{h}^{*}$. Consequently, in the equilibrium state, we assume that income and expenditure are equal and that there is only expenditure on consumption. The FIES provides an estimation of the total household expenditure $e_{h}^{FIES}$ that allows to determine the annual balance of income and expenditure of each household and its annual surplus. We assume that in the steady state every household keeps one year’s surplus as precautionary savings $s_{h}^{*}=i_{h}^{*}-e_{h}^{FIES}$, but does not accumulate further savings.

#### Recovery dynamics in the aftermath of floods

In the aftermath of a flood, the household expenditure is split into expenditure on consumption and expenditure on recovery. Each directly affected household decides upon its optimal recovery rate $\lambda_{h}$ by intertemporally maximizing its expected future well-being (Equation 3) from consumption c(t) over their planning horizon $T_{p}$ with the future discount rate ρ,

(Equation 6) $$\max_{{\left\{c_{t}^{\prime }\right\}}_{t^{\prime }=t_{shock}}^{t_{shock}+T_{p}}}W_{h}=\int_{t_{shock}}^{t_{shock}+T_{p}}u\left(c\left(t\right)\right)e^{-\rho \left(t-t_{shock}\right)}dt\;\text{subject}\;\text{to}\;c\left(t\right)\geq c_{sub}\;\text{for}\;t\in \left[t_{shock},t_{shock}+T_{p}\right]\text{.}$$

Thereby the household tries to avoid falling below the subsistence level of consumption $c_{sub}$ at any point in time $t^{\prime }\in \left[t_{shock},T_{p}\right]$ and the consumption can be written as $c\left(t\right)=c^{*}\left(t\right)-\Delta c\left(t\right)$. The total consumption losses $\Delta c_{h}\left(t\right)$ of a a household in the recovery phase (at time $t\geq t_{shock}$) equal the sum of its income losses $\Delta i_{h}\left(t\right)$ plus its spendings on asset recovery $\Delta c_{h}^{reco}\left(t\right)$ minus available precautionary savings $s_{h}\left(t\right)$,

(Equation 7) $$\Delta c_{h}\left(t\right)=\Delta i_{h}\left(t\right)+\Delta c_{h}^{reco}\left(t\right)-s_{h}\left(t\right)\text{.}$$

In the following, we derive expressions for the contributions $\Delta i_{h}\left(t\right)$ and $\Delta c_{h}^{reco}$. The spending of precautionary savings allows to smooth peak consumption losses. As the spending rate depends on the recovery pathway we discuss this in a specific section (cf. Sec. Spending of precautionary savings).

#### Consumption reduction due to recovery efforts

When a flood strikes at time $t_{shock}$, it directly affects a certain fraction of the households in the flood affected area (cf. Model calibration). Which share $\Delta k_{h}^{eff}$ of its stock of physical assets $k_{h}^{eff}\left(t\right)$ a directly affected household loses depends on the vulnerability $\nu_{h}^{*}\in \left(0,1\right)$ of its assets,

(Equation 8) $$\Delta k_{h}^{eff}\left(t_{shock}\right)=\nu_{h}^{*}k_{h}^{eff}\left(t\right)\text{.}$$

This asset vulnerability varies among households and is directly estimated from the building structure given in the FIES survey as discussed in Sec. Model Calibration. Each directly affected household is assumed to rebuild its asset stock exponentially,

(Equation 9) $$\Delta k_{h}^{eff}\left(t\right)=\Delta k_{h}^{eff}\left(t_{shock}\right)e^{-\lambda_{h}\left(t-t_{shock}\right)}\text{.}$$

Thereby, the household has to choose the rate of reconstruction $\lambda_{h}$ carefully since to finance the reconstruction efforts, it has to reduce its consumption proportionally to $\lambda_{h}$ and $\Delta k_{h}^{eff}$,

(Equation 10) $$\Delta c_{h}^{reco}\left(t\right)=-\frac{d}{dt}\left(\Delta k_{h}^{eff}\left(t\right)\right)=\lambda_{h}\Delta k_{h}^{eff}\left(t\right)\text{.}$$

#### Income losses in recovery phase

In general, the flood affects the income of a household through two impact channels. First, directly affected households perceive income losses that are proportional to their losses $\Delta k_{h}^{eff}\left(t\right)$ in productive assets (cf. Figure 2A). Second, all households that receive social transfers are affected by reductions of social spendings of the government in the disaster aftermaths. Through the second impact channel, floods also affect households which are not directly affected by the disaster. Since we assume that the government does not take up additional debt in the disaster aftermath, the reduction in social transfers is directly proportional to the national losses in productive assets $L\left(t\right)=\sum_{h=0}^{N_{h}}\omega_{h}\Delta k_{h}^{eff}\left(t\right)$ relative to the steady state value of the national stock of productive assets $K^{*}$. Combining both channels allows to express the income losses of a household as,

(Equation 11) $$\Delta i_{h}\left(t\right)=\max\left[0,\left(1-\delta_{sp}^{tax}\right)\pi \Delta k_{h}^{eff}\left(t\right)+\frac{L\left(t\right)}{K^{*}}i_{h}^{sp}\right]\text{.}$$

#### Determination of the optimal recovery rate

We assume that households do not account for disaster-induced changes in social transfers and further neglect their precautionary savings when determining the optimal reconstruction rate of the capital stock $\lambda_{h}$ by maximizing their expected well-being $W_{h}$ over their foresight horizon $T_{p}$ in the disaster aftermath. Consequently, for the optimization process, we assume income and expenditure to be equal. For the well-being maximization, we therefore set $\lambda_{sp}^{tax}=i_{h}^{sp}=s_{h}=0$. Inserting Equations 7, 8, 9, 10, and 11 into the general form of intertemporal well-being (Equation 6), then allows us to write $W_{h}$ as,

$$\begin{array}{cc} W_{h} & =\frac{{\left(k_{h}^{eff,*}\right)}^{1-\eta }}{1-\eta }\int_{t_{shock}}^{t_{shock}+T_{p}}{\left[\pi -\left(\pi +\lambda_{h}^{t1}\right)\nu_{h}e^{-\lambda_{h}^{t1}\left(t-t_{shock}\right)}\right]}^{1-\eta }e^{-\rho \left(t-t_{shock}\right)}dt\\ & =\frac{{\left(k_{h}^{eff,*}\right)}^{1-\eta }}{1-\eta }\int_{0}^{T_{p}}{\left[\pi -\left(\pi +\lambda_{h}^{t1}\right)\nu_{h}e^{-\lambda_{h}^{t1}\left(t-t_{shock}\right)}\right]}^{1-\eta }e^{-\rho \left(t-t_{shock}\right)}dt\text{.} \end{array}$$

Here, we have introduced the vulnerability $\nu_{h}$ that represents the ratio of the damaged physical asset stock directly after the shock to its steady state value, $\nu_{h}=\frac{\nu_{h}^{*}k_{h}^{eff}\left(t_{shock}\right)}{k_{h}^{eff,*}}$. The necessary condition (Euler equation) for the (unconstrained) well-being maximization then reads,

(Equation 12) $$\begin{array}{cc} \frac{\partial W}{\partial \lambda }=0\text{,} & \\ \Leftrightarrow \;0 & =\int_{0}^{T_{p}}{\left[\pi -\left(\pi +\lambda_{h}^{t1}\right)\nu_{h}e^{-\lambda_{h}^{t1}t}\right]}^{-\eta }\left(t\left(\pi +\lambda_{h}^{t1}\right)-1\right)e^{-\left(\rho +\lambda_{h}^{t1}\right)t}dt\text{.} \end{array}$$

The integral cannot be solved analytically, we determine the optimal $\lambda_{h}^{t1}$ numerically. We label this recovery process where the constraint that consumption must not fall below its subsistence level is not binding as recovery type 1 and the resulting recovery rate as $\lambda_{h}^{t1}$ (cf. Figure S3A). Beneath the type 1 recovery we consider three more recovery types that arise when the consumption constraint is binding and the household would (temporally) fall below the subsistence line when determining its asset reconstruction rate from Equation 12. To avoid falling below the subsistence line, some households have to recover at a lower pace but can always keep their consumption above subsistence level during the recovery phase (type 2 recovery), $i_{h}^{*}-\Delta i_{h}\left(t\right)-\lambda_{h}^{t1}\Delta k_{h}^{eff}<c_{sub}$ and $i_{h}^{*}-\Delta i_{h}\left(t\right)>c_{sub}$. We assume that type 2 households determine their recovery spending in every time step until exponential recovery with $\lambda_{h}^{t1}$ is possible in $t_{\exp}$. These households reduce in each time step, in which they cannot afford reconstruction with $\lambda_{h}^{t1}$, its consumption to the subsistence level less the constant recovery spending of households in subsistence $R_{sub}$ (cf. Sec. Model Calibration), and uses its remaining post-disaster income plus the constant recovery average savings rate of households in subsistence $R_{sub}$ (cf. Sec. Model calibration) to reconstruct (Figure S3B),

(Equation 13) $$\lambda_{h}^{t2}\left(t\right)=i_{h}^{*}-\Delta i_{h}\left(t\right)-c_{sub}+R_{sub}\text{.}$$

Thus, the recovery of its asset stock can be written as,

$$\Delta k_{h}^{eff}\left(t\right)=\left\{\begin{array}{cc} \Delta k_{h}^{eff,<}\left(t\right)-\lambda_{h}^{t2}\left(t\right) & \text{if}\;i_{h}^{*}-\Delta i_{h}\left(t\right)-\lambda_{h}^{t1}\Delta k_{h}^{eff}<c_{sub},\Delta k_{h}^{eff}\left(t_{\exp}\right)e^{\lambda_{h}^{t1}\left(t-t_{\exp}\right)},\\ \text{else}, & \end{array}\right)$$

where $\Delta k_{h}^{eff,<}\left(t\right)$ denotes the losses to the capital stock at the beginning of time step t. A household that falls under the subsistence line due to the shock, but usually lives above the subsistence line, $i_{h}^{*}-\Delta i_{h}\left(t\right)<c_{sub}andi_{h}^{*}>c_{sub}$, follows a type 3 recovery path consisting of three recovery modes (Figure S3C). Starting below the subsistence line, the household recovers at the standard recovery rate for people living under the subsistence line $R_{sub}$ until it reaches the subsistence line. Thus, in the case of recovery below the subsistence line we can set $\lambda_{h}^{t3}=R_{sub}$. When the subsistence level is reached at time $t_{sub}$ it recovers such as a type 2 household,

$$\Delta k_{h}^{eff}\left(t\right)=\left\{ \begin{array}{ll} \max\left[0,\Delta k_{h}^{eff}\left(t_{shock}\right)-R_{sub}\left(t-t_{shock}\right)\right] & \text{if}\;i_{h}^{*}-\Delta i_{h}\left(t\right)<c_{sub},\\ \Delta k_{h}^{eff,<}\left(t\right)-\lambda_{h}^{t2}\left(t\right) & \text{if}\;i_{h}^{*}-\Delta i_{h}\left(t\right)>c_{sub}\geq i_{h}^{*}-\Delta i_{h}\left(t\right)-\lambda_{h}^{t1}\Delta k_{h}^{eff}\left(t\right),\\ \Delta k_{h}^{eff}\left(t_{\exp}\right)e^{\lambda_{h}^{t1}\left(t-t_{\exp}\right)} & \text{if}\;i_{h}^{*}-\Delta i_{h}\left(t\right)-\lambda_{h}^{t1}\Delta k_{h}^{eff}\left(t\right)>c_{sub}. \end{array}\right.$$

A type 4 household already lives below the subsistence line in the steady state, $i_{h}^{*}<c_{sub}$. We assume that it recovers with the average savings rate for households living under the subsistence line (Figure S3D),

$$\Delta k_{h}^{eff}\left(t\right)=\max\left[0,\Delta k_{h}^{eff}\left(t_{shock}\right)-R_{sub}\left(t-t_{shock}\right)\right]\text{.}$$

#### Consumption losses smoothed by precautionary savings

During the recovery period, expenditure can exceed income as households may smooth their consumption losses by means of their precautionary savings. We assume that type 1 and type 4 households plan the spending of their precautionary savings directly after each shock and do not change it until they have recovered or a new shock arises. Type 2 and type 3 households change their recovery pathway within the recovery phase and optimize their spending of savings each time when they enter a new recovery phase. After complete recovery, households start to set aside precautionary savings until they have reached the steady state level equaling their income net of expenditure (income surplus) for one year. We assume that households regrow their precautionary savings with a monthly rate of one twelfth of this surplus so that they recover within one year if not subject to further shocks. While households regrow their savings, their income exceeds their expenditure on consumptions.

##### Type 1 recovery

If a household is on a type 1 recovery track, it can estimate its expected cumulative consumption losses and already at $t_{shock}$ and can smooth them with its precautionary savings (cf. Figure S3A). The consumption losses before smoothing are the sum of its income losses $\Delta i_{h}\left(t\right)$ (Equation 11) and its consumption losses due to recovery efforts $\Delta c_{h}^{reco}\left(t\right)$ (Equation 10). Inserting Equation 9 in Equations 10 and 11, we see that when neglecting the income from social transfers $\left(i_{h}^{sp}\right)$ household consumption in the absence of precautionary savings recovers exponentially,

(Equation 14) $$\Delta c_{h}^{ns}\left(t\right)=\Delta c_{h}^{ns}\left(t_{shock}\right)e^{-\lambda_{h}^{t1}\left(t-t_{shock}\right)}\text{,}$$

where we have introduced the consumption losses directly after the shock as $\Delta c_{h}^{ns}\left(t_{shock}\right)=\left(\left(1-\delta_{sp}^{tax}\right)\pi +\lambda_{h}^{t1}\right)\Delta k_{h}^{eff}\left(t_{shock}\right)$. By integrating Equation 14 over time, we see that in the limit of full recovery t↗∞, the cumulative expected consumption losses without savings are given by $\Sigma_{h}=\Delta c_{h}^{ns}\left(t_{shock}\right)/\lambda_{h}^{t1}$. If the cumulative expected consumption losses can be smoothed by the precautionary savings, $\Sigma_{h}\leq s_{h}\left(t_{shock}\right)$, the household employs them to compensate for consumption losses in every time-step of the recovery phase. If expected cumulative consumption losses are larger than its precautionary savings there remains a floor level of consumption losses $s_{h}^{f}>0$ that cannot be smoothed by savings. To calculate this floor level, we first invert Equation 14 to write the time spent in recovery as a function of the consumption losses,

(Equation 15) $$t\left(\Delta c_{h}^{ns}\right)=-{\left(\lambda_{h}^{t1}\right)}^{-1}\;\ln\left(\frac{\Delta c_{h}^{ns}}{\Delta c_{h}^{ns}\left(t_{shock}\right)}\right)\text{.}$$

Equating $s_{h}\left(t_{shock}\right)$ to integrated consumption losses up to $s_{h}^{f,1}$, yields an implicit analytic expression for $s_{h}^{f,1}$,

(Equation 16) $$\begin{array}{cc} s_{h}\left(t_{shock}\right) & =\int_{s_{h}^{f,1}}^{\Delta c_{h}^{ns}\left(t_{shock}\right)}t\left(\Delta c_{h}^{ns}\right)d\Delta c_{h}^{ns}=-{\left(\lambda_{h}^{t1}\right)}^{-1}\int_{s_{h}^{f,1}}^{\Delta c_{h}^{ns}\left(t_{shock}\right)}\;\ln\left(\frac{\Delta c_{h}^{ns}}{\Delta c_{h}^{ns}\left(t_{shock}\right)}\right)d\Delta c_{h}^{ns}\\ \Leftrightarrow 0 & =\frac{1}{\lambda_{h}^{t1}}\left(\Delta c_{h}^{ns}\left(t_{shock}\right)-s_{h}^{f,1}\left(\ln\left(\frac{\Delta c_{h}^{ns}\left(t_{shock}\right)}{s_{h}^{f,1}}\right)+1\right)\right)-s_{h}\left(t_{shock}\right)\text{.} \end{array}$$

Finally, the resulting consumption losses under type 1 recovery accounting for smoothing with precautionary savings can be written as,

(Equation 17) $$\Delta c_{h}\left(t\right)=\left\{ \begin{array}{ll} \min\left[s_{h}^{f,1},\Delta c_{h}^{ns}\left(t_{shock}\right)e^{-\lambda_{h}^{t1}\left(t-t_{shock}\right)}\right] & \text{for}\;\Sigma_{h}>s_{h}\left(t_{shock}\right),\\ 0 & \text{else}. \end{array}\right.$$

##### Type 2 recovery

In the quasi-linear part of the type 2 recovery, households update their recovery rate $\lambda_{h}^{t2}$ in each point in time according to Equation 13. However, since households cannot foresee future changes in their recovery rate $\lambda_{h}^{t2}$, they form their expectation on the recovery of their asset, consumption, and income losses and assume that their recovery rate will remain at the present level $\lambda_{h}^{t2}\left(t\right)=\lambda_{h}^{t2}\left(t_{reco}\right)\;\text{for}\;t>t_{reco}$. From Equation 13, we see that this allows to write their expected asset damages as a linear function of time $\Delta k_{h}^{eff}\left(t\right)=\Delta k_{h}^{eff}\left(t_{reco}\right)-\lambda_{h}^{t2}\left(t_{reco}\right)\left(t-t_{reco}\right)\;\text{for}\;t\geq t_{reco}$. From Equations 10 and 11 it then follows that for $t>t_{reco}$ expected consumption losses from reconstruction and income losses can be expressed as $\Delta c_{h}^{reco}\left(t\right)=\lambda_{h}^{t2}\left(t_{reco}\right)$ and $\Delta i_{h}\left(t\right)=\max\left[0,\pi \left(1-\delta_{sp}^{tax}\right)\left(\Delta k_{h}^{eff}\left(t_{reco}\right)-\lambda_{h}^{t2}\left(t_{reco}\right)\left(t-t_{reco}\right)\right)\right]$, respectively, where we have set $i_{h}^{sp}=0$ in the equation for the expected consumption losses (cf. type 1 recovery). According to Equation 7, the total consumption losses that households expects at time $t_{reco}$ for any future time step of the quasi-linear reconstruction phase before smoothing by precautionary savings $\Delta i_{h}\left(t\right)+\Delta c_{h}^{reco}\left(t\right)$ (cf. Equation 7) can be expressed as

(Equation 18) $$\Delta c_{h}^{ns,2}\left(t\right)=\max\left[0,\Delta c_{h}^{ns,2}\left(t_{reco}\right)-\pi \lambda_{h}^{t2}\left(t_{reco}\right)\left(1-\delta_{sp}^{tax}\right)\left(t-t_{reco}\right)\right]\text{.}$$

The inverse relationship then reads,

(Equation 19) $$t\left(\Delta c_{h}^{ns,2}\right)=\frac{\Delta c_{h}^{ns,2}\left(t_{reco}\right)-\Delta c_{h}^{ns,2}}{\pi \left(1-\delta_{sp}^{tax}\right)\;\lambda_{h}^{t2}\left(t_{reco}\right)}\text{.}$$

We now can calculate the floor level of consumption that remains after smoothing with precautionary savings, analogously to the type 1 recovery (cf. Equation 16), yielding the following quadratic relation for $s_{h}^{f,2}$,

(Equation 20) $$0=\frac{s_{h}^{f,2^{2}}}{2}-s_{h}^{f,2}\Delta c\left(t_{reco}\right)+\frac{\Delta c{\left(t_{reco}\right)}^{2}}{2}-\pi \lambda_{h}^{t2}\left(t_{reco}\right)\left(1-\delta_{sp}^{tax}\right)\;s_{h}\left(t_{reco}\right)\text{.}$$

Solving for $s_{h}^{f,2}\left(t_{reco}\right)$ and choosing “-” branch then allows to write $s_{h}^{f,2}\left(t_{reco}\right)$ as,

(Equation 21) $$s_{h}^{f,2}\left(t_{reco}\right)=\Delta c_{h}^{ns}\left(t_{reco}\right)-\sqrt{2\pi \lambda_{h}^{t2}\left(t_{reco}\right)\left(1-\delta_{sp}^{tax}\right)\;s_{h}\left(t_{reco}\right)}\text{.}$$

Again, it is important to note that the floor level is calculated in each time-step of the quasi-linear recovery phase, which is why $s_{h}^{f,2}$ is time dependent. The solution for the exponential phase of the type 2 recovery can be obtained as for a type 1 household. Thus, the recovery path of the consumption of a type 2 household can be written as,

(Equation 22) $$\Delta c_{h}\left(t\right)=\left\{ \begin{array}{ll} \max\left[s_{h}^{f,2}\left(t\right),0\right] & \text{if}\;i_{h}^{*}-\Delta i_{h}\left(t\right)-\lambda_{h}^{t1}\Delta k_{h}^{eff}\left(t\right)<c_{sub},\\ \min\left[s_{h}^{f,1},\Delta c_{h}^{ns}\left(t_{\exp}\right)e^{-\lambda_{h}^{t1}\left(t-t_{\exp}\right)}\right] & \text{if}\;\Sigma_{h}>s_{h}\left(t_{\exp}\right)\;\text{and}\;i_{h}^{*}-\Delta i_{h}\left(t\right)-\lambda_{h}^{t1}\Delta k_{h}^{eff}\left(t\right)>c_{sub},\\ 0 & \text{else}, \end{array}\right.$$

where $t_{\exp}$ is the point in time from which type 1 recovery is possible (cf. Equation 14).

##### Type 3 recovery

Households of recovery type 3 start their recovery below the subsistence line where they recover with a constant rate $\lambda_{h}^{t2}=R_{sub}$. In this phase they do not experience additional consumption losses due to recovery efforts as $R_{sub}$ is assumed to be the amount they save in normal times. Thus, $\Delta c_{h}^{reco}\left(t\right)=0$ and their expected non-smoothed consumption losses at any time $t\geq t_{shock}$ of the recovery phase can be written as,

(Equation 23) $$\Delta c_{h}^{ns,3}\left(t\right)=\Delta i_{h}\left(t\right)=\max\left[0,\pi \left(1-\delta_{sp}^{tax}\right)\left(\Delta k_{h}^{eff}\left(t_{shock}\right)-R_{sub}\;\left(t-t_{shock}\right)\right)\right]\text{.}$$

The solution for the floor consumption level in this phase $s_{h}^{f3}$, can be derived analogously the relation obtained for the quasi-linear recovery phase of a type 2 household (Equations 18, 19, 20, and 21) The only difference is that $R_{sub}$ is in contrast to $\lambda_{h}^{t2}$ not time dependent. In consequence, the floor level of consumption is constant throughout the recovery phase,

(Equation 24) $$s_{h}^{f,3}=\Delta c_{h}^{ns,3}\left(t_{shock}\right)-\sqrt{2\pi R_{sub}\left(1-\delta_{sp}^{tax}\right)\;s_{h}\left(t_{shock}\right)}\text{.}$$

Since the second and third phase of a type 3 recovery correspond to type 2 recovery, the consumption losses in the recovery phase of type 3 household after smoothing with its precautionary savings can be written as

$$\Delta c_{h}\left(t\right)=\left\{ \begin{array}{ll} \min\left[s_{h}^{f,3}\left(t\right),\Delta c_{h}^{ns,3}\left(t\right)\right] & \text{if}\;\Sigma_{h}>s_{h}\left(t_{shock}\right)\;\text{and}\;i_{h}^{*}-\Delta i_{h}\left(t\right)<c_{sub},\\ \max\left[s_{h}^{f,2}\left(t\right),0\right] & \text{if}\;i_{h}^{*}-\Delta i_{h}\left(t\right)>c_{sub}\geq i_{h}^{*}-\Delta i_{h}\left(t\right)-\lambda_{h}^{t1}\Delta k_{h}^{eff}\left(t\right),\\ \min\left[s_{h}^{f,1},\Delta c_{h}^{ns}\left(t_{\exp}\right)e^{-\lambda_{h}^{t1}\left(t-t_{\exp}\right)}\right] & \text{if}\;\Sigma_{h}>s_{h}\left(t_{\exp}\right)\;\text{and}\;i_{h}^{*}-\Delta i_{h}\left(t\right)-\lambda_{h}^{t1}\Delta k_{h}^{eff}\left(t\right)>c_{sub},\\ 0 & \text{else}. \end{array}\right.$$

##### Type 4 recovery

Type 4 households always live under the subsistence line. This is why their recovery is analogous to the first recovery phase of a type 3 household. Thus, their recovery of consumption after smoothing with precautionary savings reads,

$$\Delta c_{h}\left(t\right)=\left\{ \begin{array}{ll} \min\left[s_{h}^{f,3},\Delta c_{h}^{ns,3}\left(t\right)\right] & \text{for}\;\Sigma_{h}>s_{h}\left(t_{shock}\right),\\ 0 & \text{else}. \end{array}\right.$$

#### Calculation of well-being losses

Finally, we derive the households’ total accumulated well-being losses from Equation 10 by subtracting the accumulated utility gained under reduced consumption from an unperturbed consumption pathway, $\Delta W_{h}\left(t_{sim}\right)=\frac{1}{1-\eta }\int_{0}^{tsim}\left[{\left(c_{h}^{*}\right)}^{1-\eta }-c_{h}{\left(t\right)}^{1-\eta }\right]dt=\frac{1}{1-\eta }\left[{\left(c_{h}^{*}\right)}^{1-\eta }t_{sim}-\int_{0}^{tsim}c_{h}{\left(t\right)}^{1-\eta }dt\right]$. For the calculation of overall well-being losses $t_{sim}$ equals the full runtime of the model, and we set the future discount rate ρ introduced in Equation 6 to zero because otherwise we would give greater relevance to earlier shocks. In some cases not all of the savings are spent on the first shock, as households cannot plan with the recovery of income from social transfers $i_{sp}$, but only for the recovery of their own asset stock. Well-being losses are often provided in monetary terms (^20^). In this work, we avoid presenting well-being losses in monetary terms, as the long runtime of the model (Table 1) produces high well-being losses compared to total asset losses, which are only considered between 2000-2018. Nevertheless, for an adequate comparison of factual and counterfactual runs, the long runtime is required. Showing both metrics in monetary terms would be misleading and we here provide well-being losses only for the comparison of impacts between factual and counterfactual scenarios and between income deciles. Well-being loss units (WBLU) express the aggregated utility loss of the household, derived from a comparison of the unperturbed consumption path and the consumption under recovery.

#### Calculation of socioeconomic resilience

The socioeconomic resilience of a household is expressed by the ratio of asset damages to well-being losses.^20^ Therefore, we need to express the well-being losses in monetary terms. To this end we introduce the consumption losses equivalent as the consumption losses that an individual earning the national mean income would suffer,

$$\Delta C_{h}^{equ}=\frac{\Delta W_{h}}{W^{\prime }},\text{with}\;W^{\prime }=\frac{\partial W}{\partial c}{|}_{av}=\frac{\partial }{\partial c}{|}_{av}\frac{c^{1-\eta }}{1-\eta }={\left(c_{av}\right)}^{-\eta }\text{,}$$

where subscript $c_{av}$ denotes the national average per-capita consumption. Thus, if a disaster causes 1 PHL Peso in well-being losses, it means that its wellbeing impact is equivalent to a 1 PHL Peso decrease in the consumption of the average Filipino. This allows us to express well-being losses, like asset damage, in monetary terms and use both metrics to define the household’s socio-economic resilience as,

$$R_{h}^{soc}=\frac{\sum_{h=0}^{n_{h}}\sum_{shock=0}^{n_{shocks}}w_{h}\Delta k_{h}^{eff}\left(t_{shock}\right)}{\Delta C_{h}^{equ}}\text{.}$$

#### Model calibration

In Table 1, we provide an overview of all the parameters calibrated to run the model. The number of flood shocks, their time distance and the number of affected households is calibrated to the number of affected people given in EM-DAT as described in Sec. Derivation of flood forcing. In the absence of shocks, the model is in a steady state. The steady state values of the key household variables income from productive assets $i_{h}^{*}$, income form social spendings $i_{h}^{sp,*}$, and precautionary savings $s_{h}^{*}$ are taken from the income and expenditure data of the FIES survey. The subsistence line assumed throughout the simulations corresponds to the international subsistence level of 350 USD, while the lower estimate corresponds to 250 USD and the upper estimate to 400 USD applying the exchange rate from 2015.^103^ We also estimate the recovery spending of people living under the subsistence line $R_{sub}$ from the FIES by calculating the average annual savings, as difference between income and expenditure, of all households living under the subsistence line. Additionally, for each household, we estimate its asset vulnerability $\nu_{h}$^20^ according to the building structure given in the FIES. The survey distinguishes between robust, moderate, and fragile buildings. We assume that this building quality is valid for all of the household’s assets and assign corresponding vulnerabilities, for each household we simulate a random variation within the uncertainty range around the basic vulnerability factor (Table 1). For the standard simulation, we derive $R_{sub}$ only from the households living below the subsistence line that have an income surplus, while households whose expenditure exceeds income are excluded. The lower estimate used for robustness checks (Figures S9, S13, and S17) of $R_{sub}$ is the average over the annual surplus from all households below the subsistence line. The higher estimate corresponds to a significantly faster recovery rate than extractable from the FIES. The planning horizon of households is set to 15 years in the main simulation, but to test the sensitivity of the results to this parameter, we also generate results for a planning horizon $T_{p}$ of 10 years and 20 years (Figures S7, S11, and S15). In the main analysis, we assume a constant productivity of capital stock π of 0.33, while we provide a sensitivity analysis for π=0.2 and π=0.45 (Figures S8, S12, and S16).
