# Supplementary material for: Understanding the distributional effects of recurrent floods in the Philippines
Source: iScience. 2025 Jan 3;28(2):111733. doi: 10.1016/j.isci.2024.111733 (PMC11803224; doi:10.1016/j.isci.2024.111733)
Supplement: Document S1. Figures S1–S18 and Tables S1 and S2 [file mmc1.pdf]

iScience, Volume *28*

Supplemental information

Understanding the distributional effects of recurrent floods in the Philippines

Inga J. Sauer, Brian Walsh, Katja Frieler, David N. Bresch, and Christian Otto

Contents

1 Supplementaty Figures 2
2 Supplementaty Tables 21
References 23

1 Supplementaty Figures

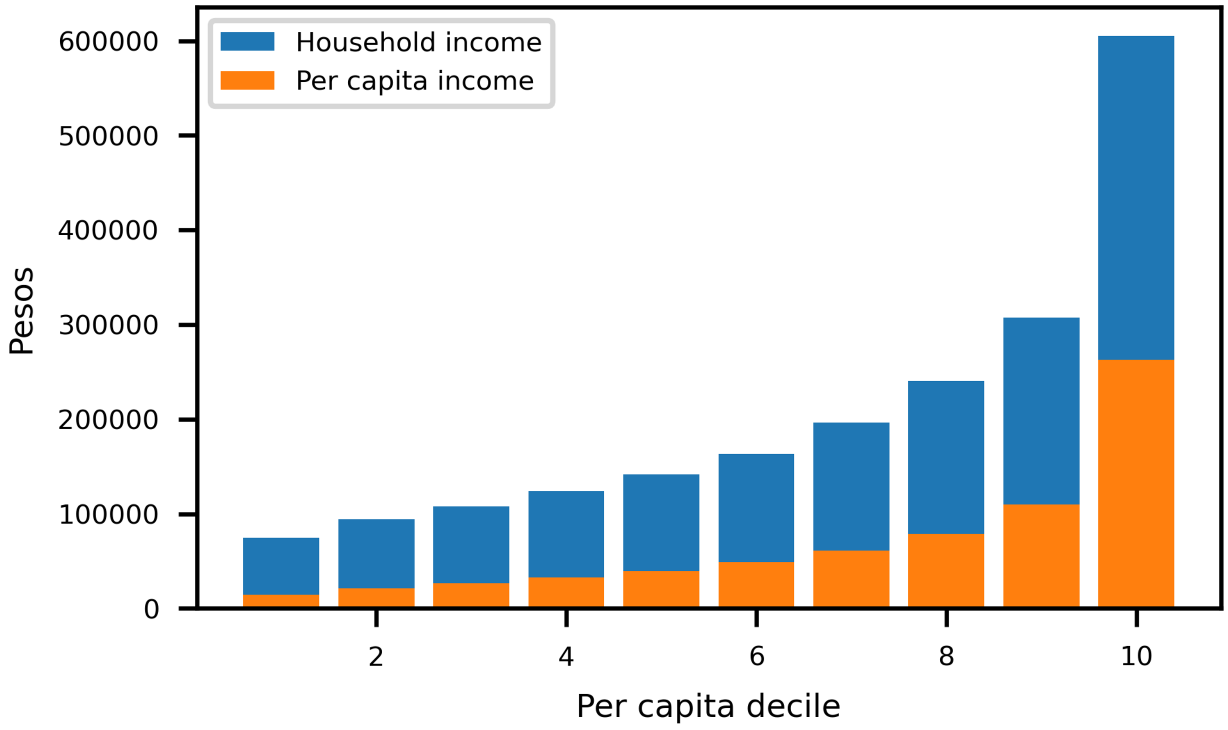

Figure S1. Income distribution across deciles. Annual average household (blue) and per capita (orange) income across deciles as recorded in the Family Income and Expenditure Survey 2015 (FIES)[1].

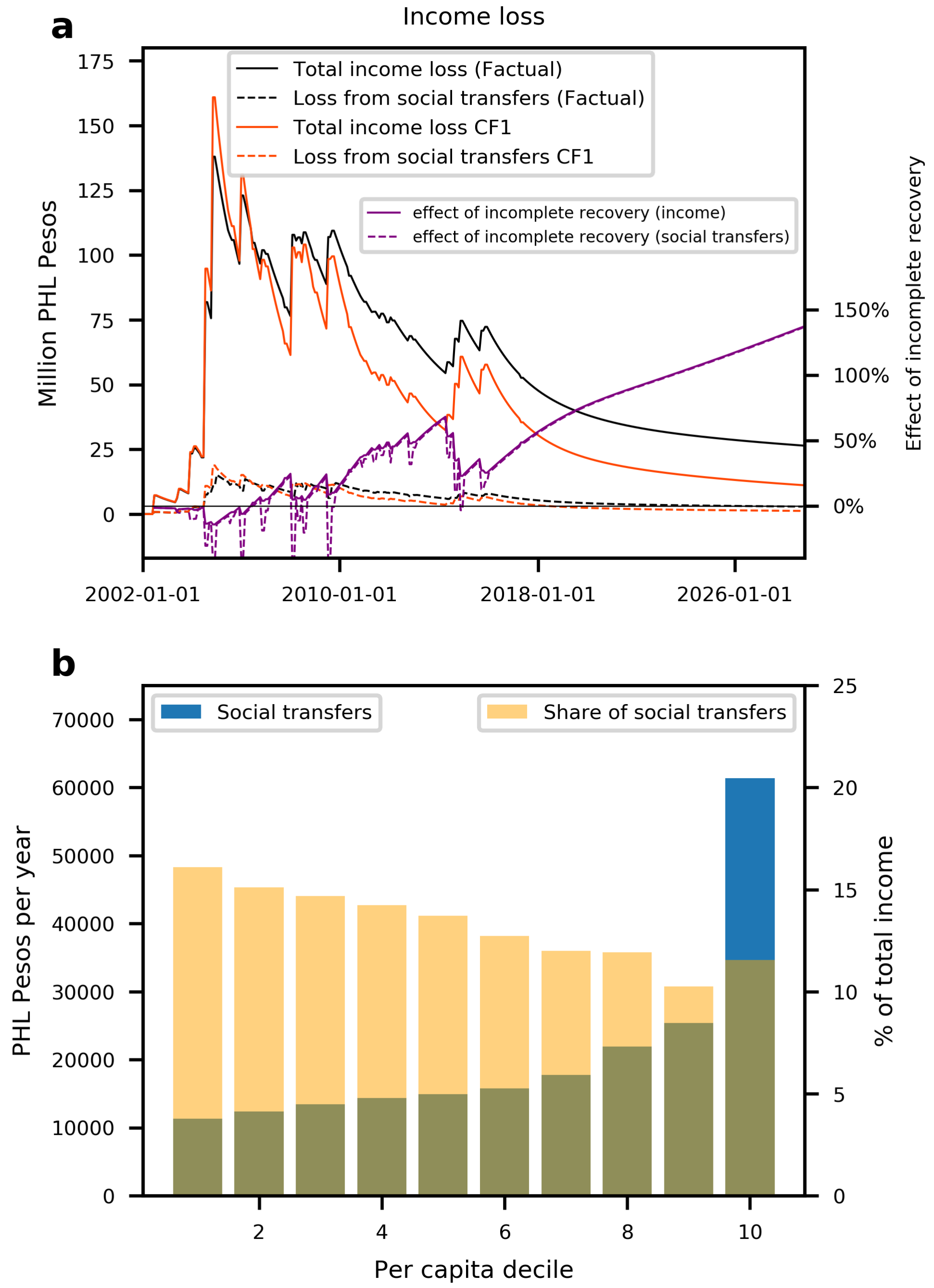

Figure S2. Income loss from social transfers. **a** Total national income loss over time composed by income loss from lost assets and income loss due to reduced social transfers and the increase of income loss caused by incomplete recovery. **b** Average income from social transfers in absolute numbers of PHL Pesos (blue) and share of social transfers of total income in percent (orange).

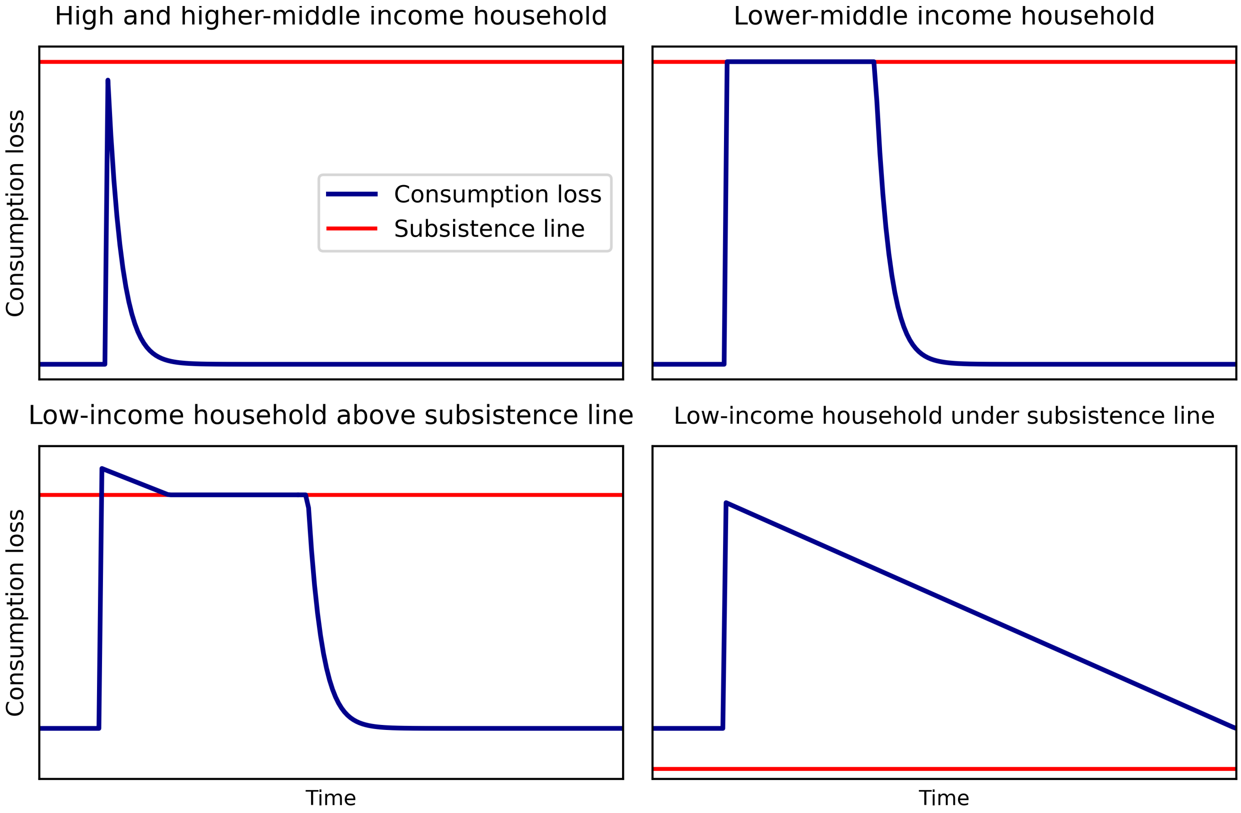

Figure S3. Recovery dynamics of consumption for different income levels. **a** Recovery type 1 - Recovery above subsistence line: The household lives usually far above subsistence line (red line - consumption loss that can be compensated without falling under subsistence line), after a shock it can recover at an exponential rate λ_h^{t1} (well-being loss is minimized). **b** Recovery type 2 - Recovery along subsistence line: The household lives usually above subsistence line (red line - consumption loss that can be compensated without living under subsistence line), after a shock it cannot recover at an exponential rate, as the household cannot afford the rate without crossing subsistence line. The household spends as much as possible without falling under subsistence line in each time step until exponential recovery is possible. **c** Recovery type 3 - Recovery starting under subsistence line: The household lives usually above subsistence line (red line - consumption loss that can be compensated without living under subsistence line), but income is reduced through the shock, so that it starts recovery under the subsistence line. It can only recover with the basic savings rate for people living under subsistence line R_{sub} until it crosses the subsistence line. After crossing the subsistence line it recovers like a household of recovery type 2. **d** Recovery type 4 - Recovery: The household lives always under subsistence line (red line - consumption loss that can be compensated without living under subsistence line, in this case it is negative meaning that the household would need additional consumption to live above subsistence line). It can only recover with the basic savings rate R_{sub} for people under subsistence line over the entire recovery period.

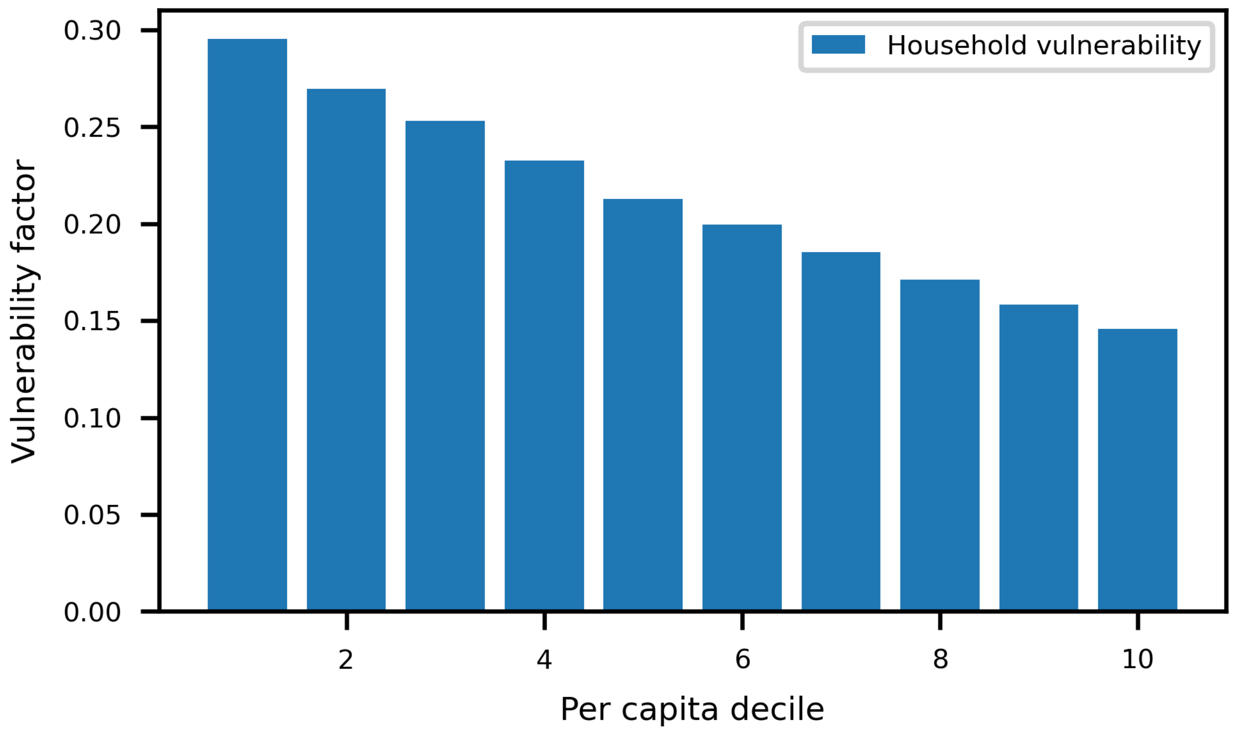

Figure S4. Income dependence of asset vulnerability. Average asset vulnerability of each income group estimated from the building structure given in the FIES survey (cf. Tab. S1)[1].

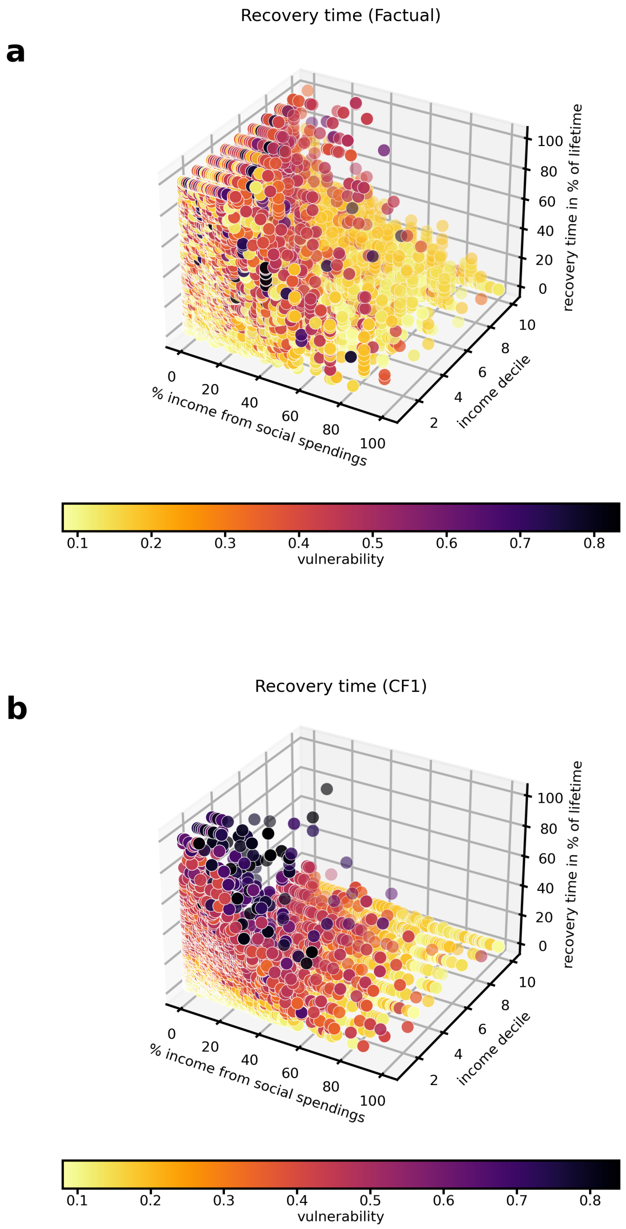

Figure S5. Recovery time. Recovery time of households plotted against income group and income share from social transfers for **a** the factual scenario and **b** counterfactual scenario 1. Colors indicate the household vulnerability factor.

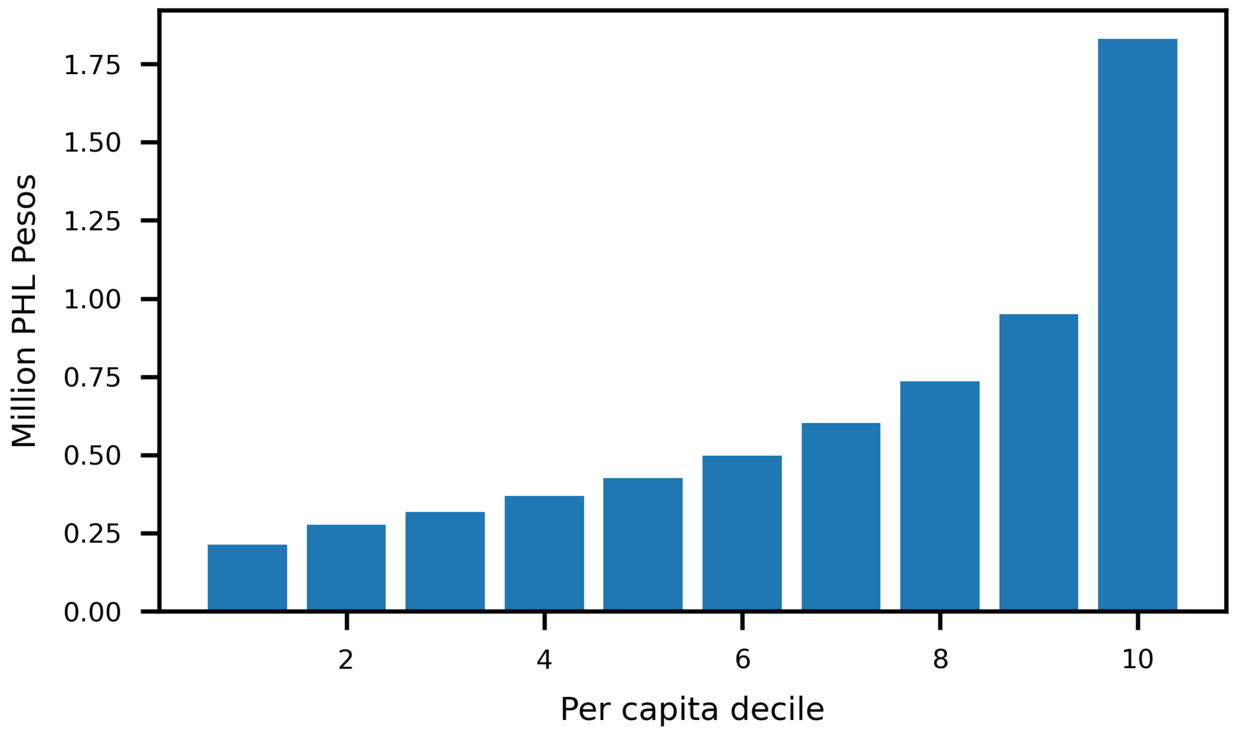

Figure S6. Average assets per income group. Average assets per household in million PHL Pesos estimated from income given in the FIES[1] and average productivity of capital stock π.

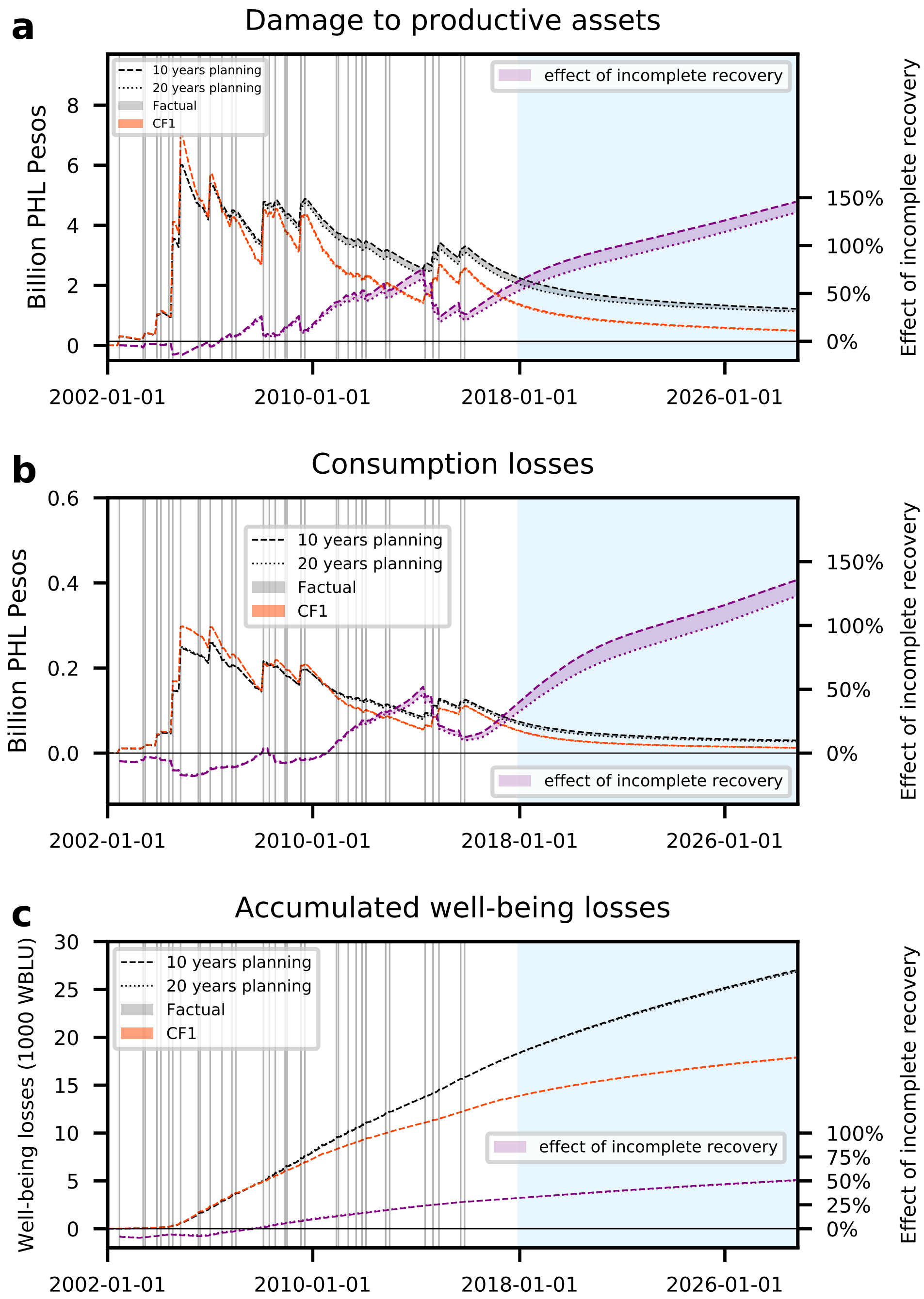

Figure S7. Nationally aggregated response dynamics to the observed flood sequence for different planning horizons T_p. **a** Temporary evolution of the damage to the stock of productive assets, **b** consumption losses, and **c** accumulated well-being losses aggregated over all households to the national level for subsequent flood shock over the period 2000-2018 covered by the Global Flood Database[2]. The black and red line indicate the factual scenario where the same households can be affected by several floods and a counterfactual scenario where households are affected only once, respectively. Gray vertical lines indicate flood events as recorded in the Global Flood Database from January 1, 2002 to January 1, 2018 (white background). The recovery phase where no further shocks are recorded as the time period is no longer covered by the Global Flood Database is indicated in blue. Dash-dotted lines in **c** indicate the relative difference between the well-being losses for the factual and the respective counterfactual scenarios. Well-being losses are measured in well-being loss units (WBLU) (Methods).

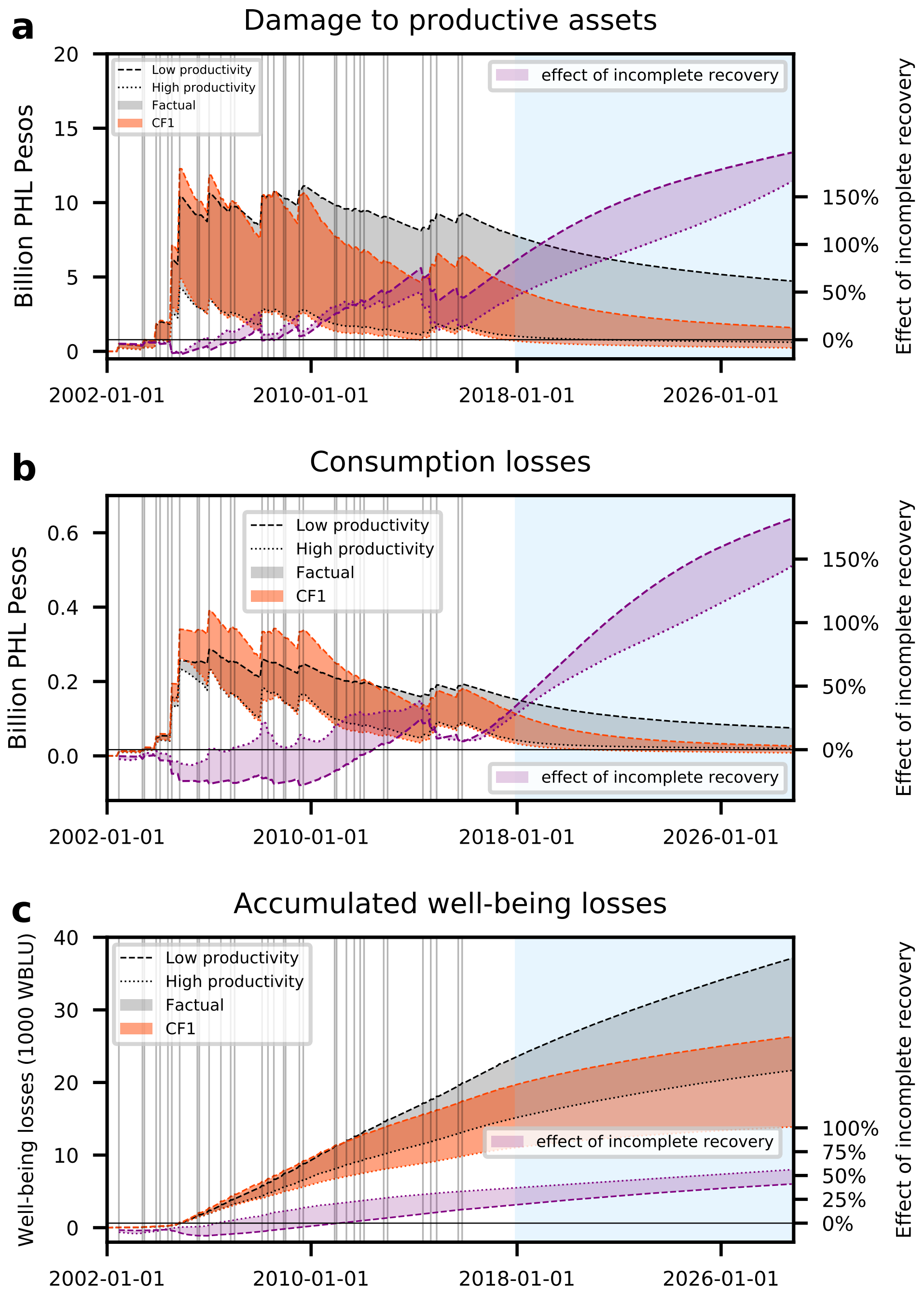

Figure S8. Nationally aggregated response dynamics to the observed flood sequence under varying productivity of capital π. **a** Temporary evolution of the damage to the stock of productive assets, **b** consumption losses, and **c** accumulated well-being losses aggregated over all households to the national level for subsequent flood shock over the period 2000-2018 covered by the Global Flood Database[2]. The black and red line indicate the factual scenario where the same households can be affected by several floods and a counterfactual scenario where households are affected only once, respectively. Gray vertical lines indicate flood events as recorded in the Global Flood Database from January 1, 2002 to January 1, 2018 (white background). The recovery phase where no further shocks are recorded as the time period is no longer covered by the Global Flood Database is indicated in blue. Dash-dotted lines in **c** indicate the relative difference between the well-being losses for the factual and the respective counterfactual scenarios. Well-being losses are measured in well-being loss units (WBLU) (Methods).

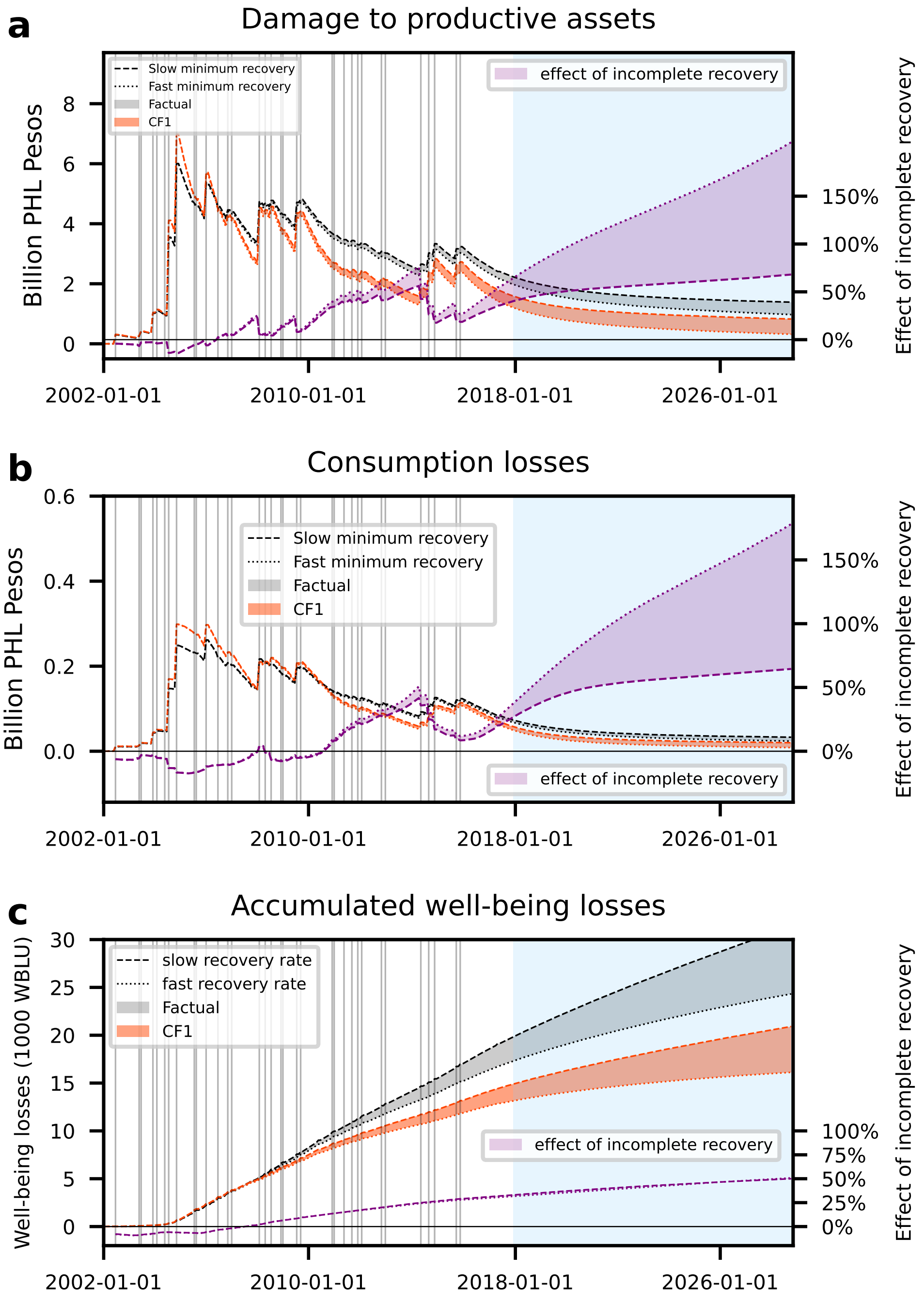

Figure S9. Nationally aggregated response dynamics to the observed flood sequence for different minimum recovery rates R_{sub}. **a** Temporary evolution of the damage to the stock of productive assets, **b** consumption losses, and **c** accumulated well-being losses aggregated over all households to the national level for subsequent flood shock over the period 2000-2018 covered by the Global Flood Database[2]. The black and red line indicate the factual scenario where the same households can be affected by several floods and a counterfactual scenario where households are affected only once, respectively. Gray vertical lines indicate flood events as recorded in the Global Flood Database from January 1, 2002 to January 1, 2018 (white background). The recovery phase where no further shocks are recorded as the time period is no longer covered by the Global Flood Database is indicated in blue. Dash-dotted lines in **c** indicate the relative difference between the well-being losses for the factual and the respective counterfactual scenarios. Well-being losses are measured in well-being loss units (WBLU) (Methods).

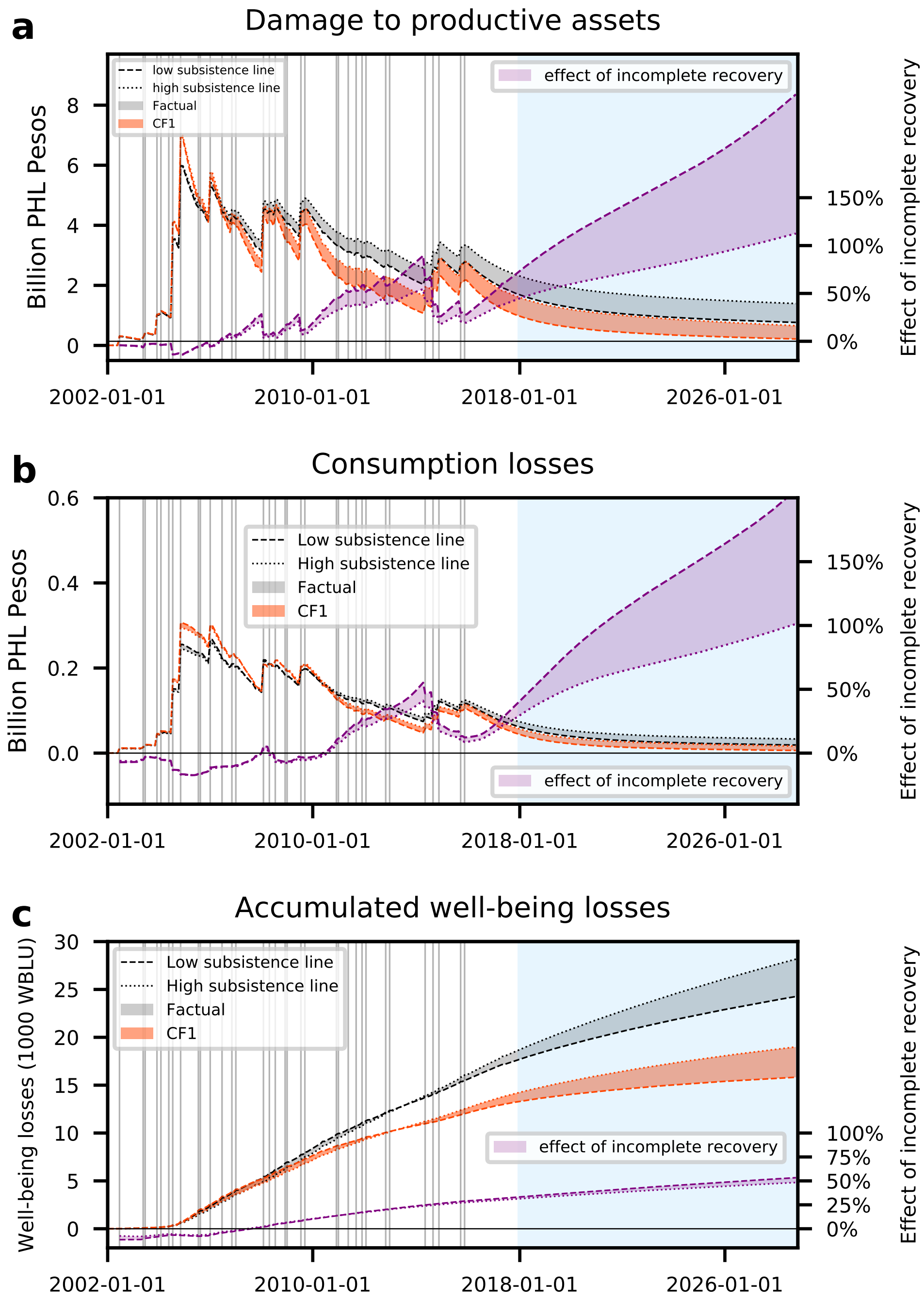

Figure S10. Nationally aggregated response dynamics to the observed flood sequence for different subsistence lines. **a** Temporary evolution of the damage to the stock of productive assets, **b** consumption losses, and **c** accumulated well-being losses aggregated over all households to the national level for subsequent flood shock over the period 2000-2018 covered by the Global Flood Database[2]. The black and red line indicate the factual scenario where the same households can be affected by several floods and a counterfactual scenario where households are affected only once, respectively. Gray vertical lines indicate flood events as recorded in the Global Flood Database from January 1, 2002 to January 1, 2018 (white background). The recovery phase where no further shocks are recorded as the time period is no longer covered by the Global Flood Database is indicated in blue. Dash-dotted lines in **c** indicate the relative difference between the well-being losses for the factual and the respective counterfactual scenarios. Well-being losses are measured in well-being loss units (WBLU) (Methods).

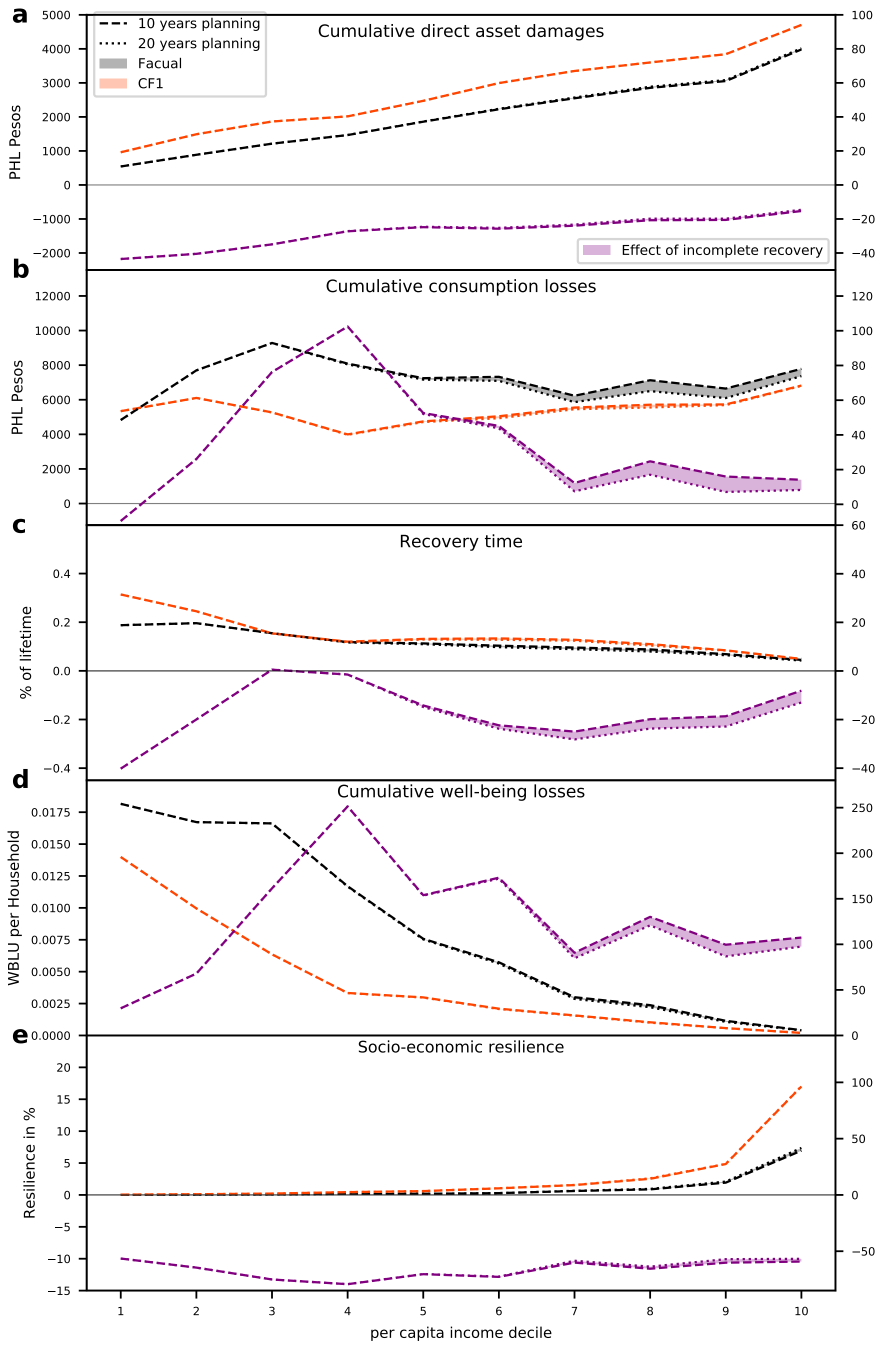

Figure S11. Distributional impacts of recurrent flood shocks for different planning horizons T_p. National averages for each per-capita income decile of the population for the factual scenario where the same household can be affected multiple times (solid black lines) and the counterfactual scenario 1 where each household can be affected at most once (solid orange lines). Absolute (left y-achses) and relative differences (right y-achses) between the factual and the counterfactual scenarios as they arise from incomplete recovery in between events in the factual scenario are denoted by dashed purple lines. Average cumulative direct asset damages **(panel a)**, average cumulative consumption losses **(panel b)**, average share of their lifetime flood affected households in the Philippines spend recovering their damaged assets from recurrent flood shocks over the period 2000-2018 **(panel c)**, average cumulative well-being losses measured in well-being loss units (WBLU) **(panel d)** and average socioeconomic resilience **(panel e)**.

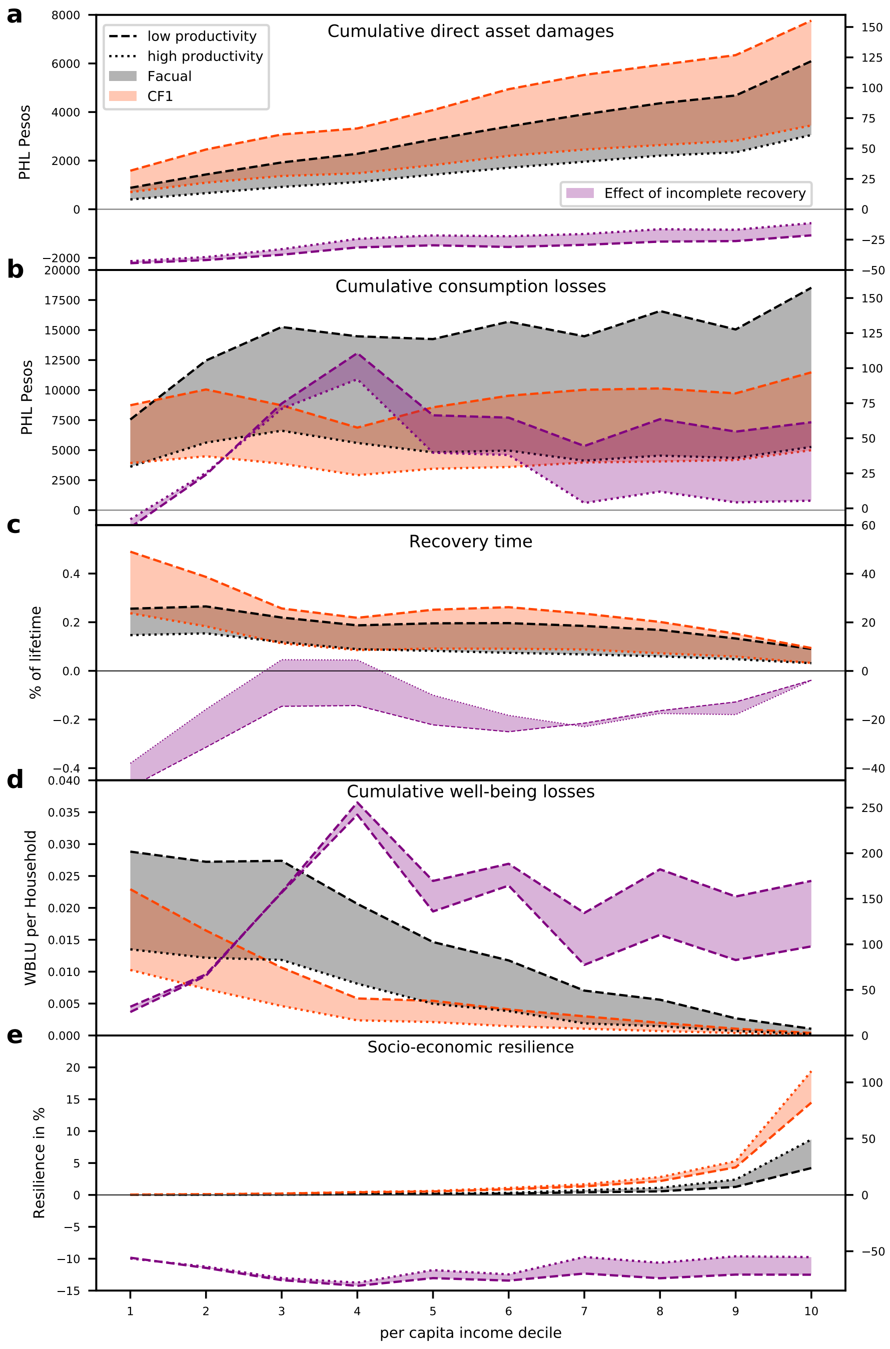

Figure S12. Distributional impacts of recurrent flood shocks under varying productivity of capital π. National averages for each per-capita income decile of the population for the factual scenario where the same household can be affected multiple times (solid black lines) and the counterfactual scenario 1 where each household can be affected at most once (solid orange lines). Absolute (left y-achses) and relative differences (right y-achses) between the factual and the counterfactual scenarios as they arise from incomplete recovery in between events in the factual scenario are denoted by dashed purple lines. Average cumulative direct asset damages **(panel a)**, average cumulative consumption losses **(panel b)**, average share of their lifetime flood affected households in the Philippines spend recovering their damaged assets from recurrent flood shocks over the period 2000-2018 **(panel c)**, average cumulative well-being losses measured in well-being loss units (WBLU) **(panel d)** and average socioeconomic resilience **(panel e)**.

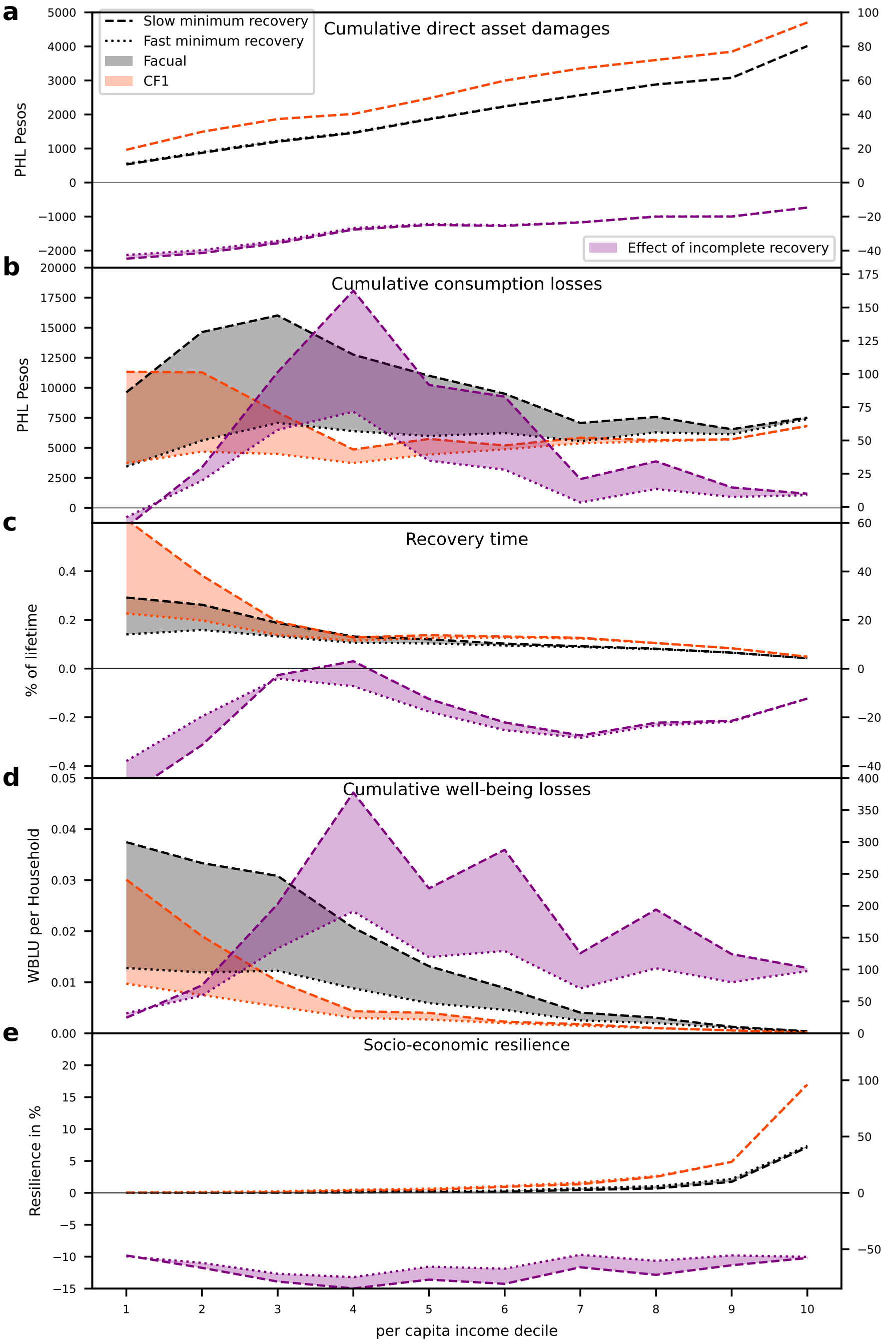

Figure S13. Distributional impacts of recurrent flood shocks for different minimum recovery rates R_{sub}. National averages for each per-capita income decile of the population for the factual scenario where the same household can be affected multiple times (solid black lines) and the counterfactual scenario 1 where each household can be affected at most once (solid orange lines). Absolute (left y-achses) and relative differences (right y-achses) between the factual and the counterfactual scenarios as they arise from incomplete recovery in between events in the factual scenario are denoted by dashed purple lines. Average cumulative direct asset damages **(panel a)**, average cumulative consumption losses **(panel b)**, average share of their lifetime flood affected households in the Philippines spend recovering their damaged assets from recurrent flood shocks over the period 2000-2018 **(panel c)**, average cumulative well-being losses measured in well-being loss units (WBLU) **(panel d)** and average socioeconomic resilience **(panel e)**.

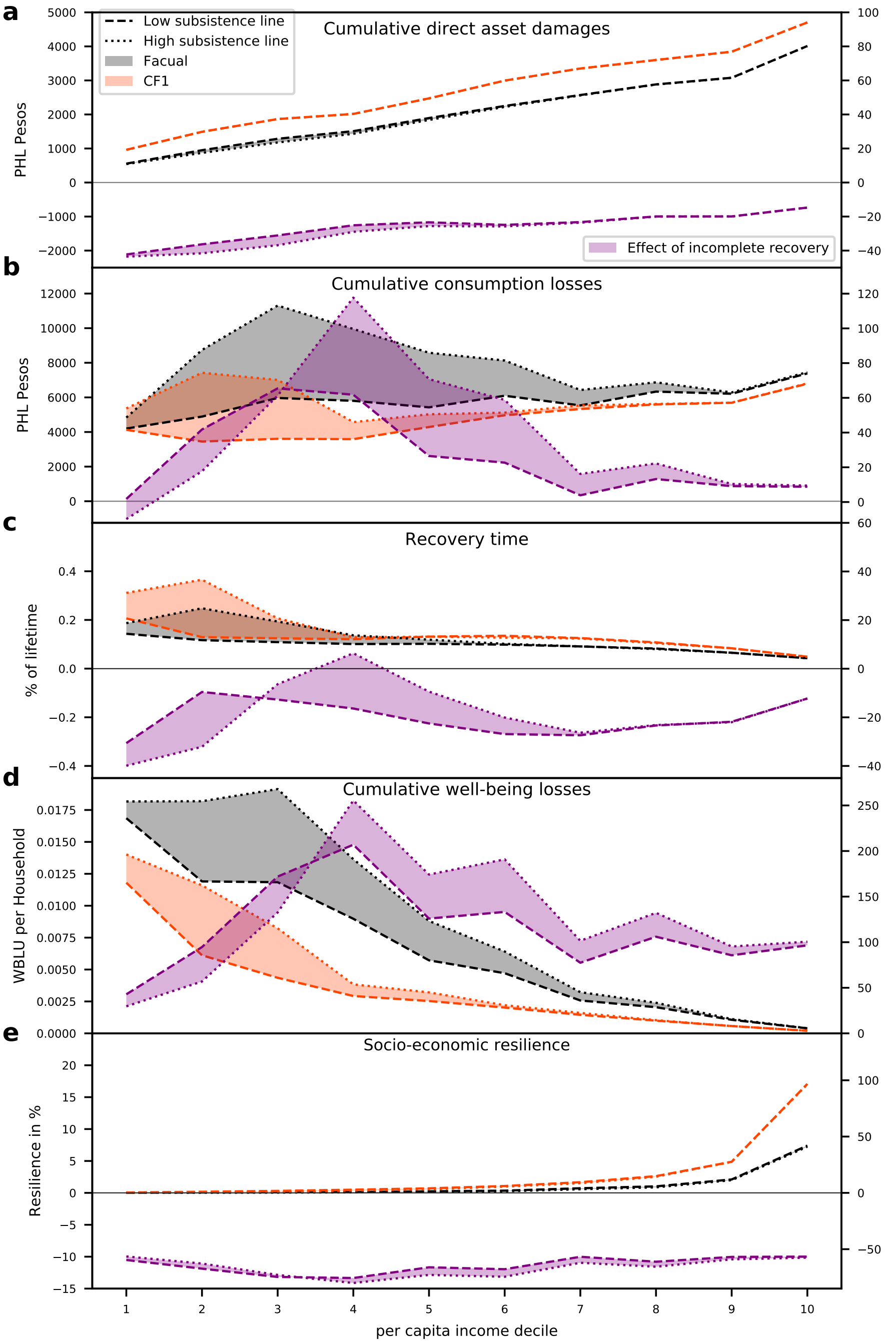

Figure S14. Distributional impacts of recurrent flood shocks for different subsistence lines. National averages for each per-capita income decile of the population for the factual scenario where the same household can be affected multiple times (solid black lines) and the counterfactual scenario 1 where each household can be affected at most once (solid orange lines). Absolute (left y-achses) and relative differences (right y-achses) between the factual and the counterfactual scenarios as they arise from incomplete recovery in between events in the factual scenario are denoted by dashed purple lines. Average cumulative direct asset damages **(panel a)**, average cumulative consumption losses **(panel b)**, average share of their lifetime flood affected households in the Philippines spend recovering their damaged assets from recurrent flood shocks over the period 2000-2018 **(panel c)**, average cumulative well-being losses measured in well-being loss units (WBLU) **(panel d)** and average socioeconomic resilience **(panel e)**.

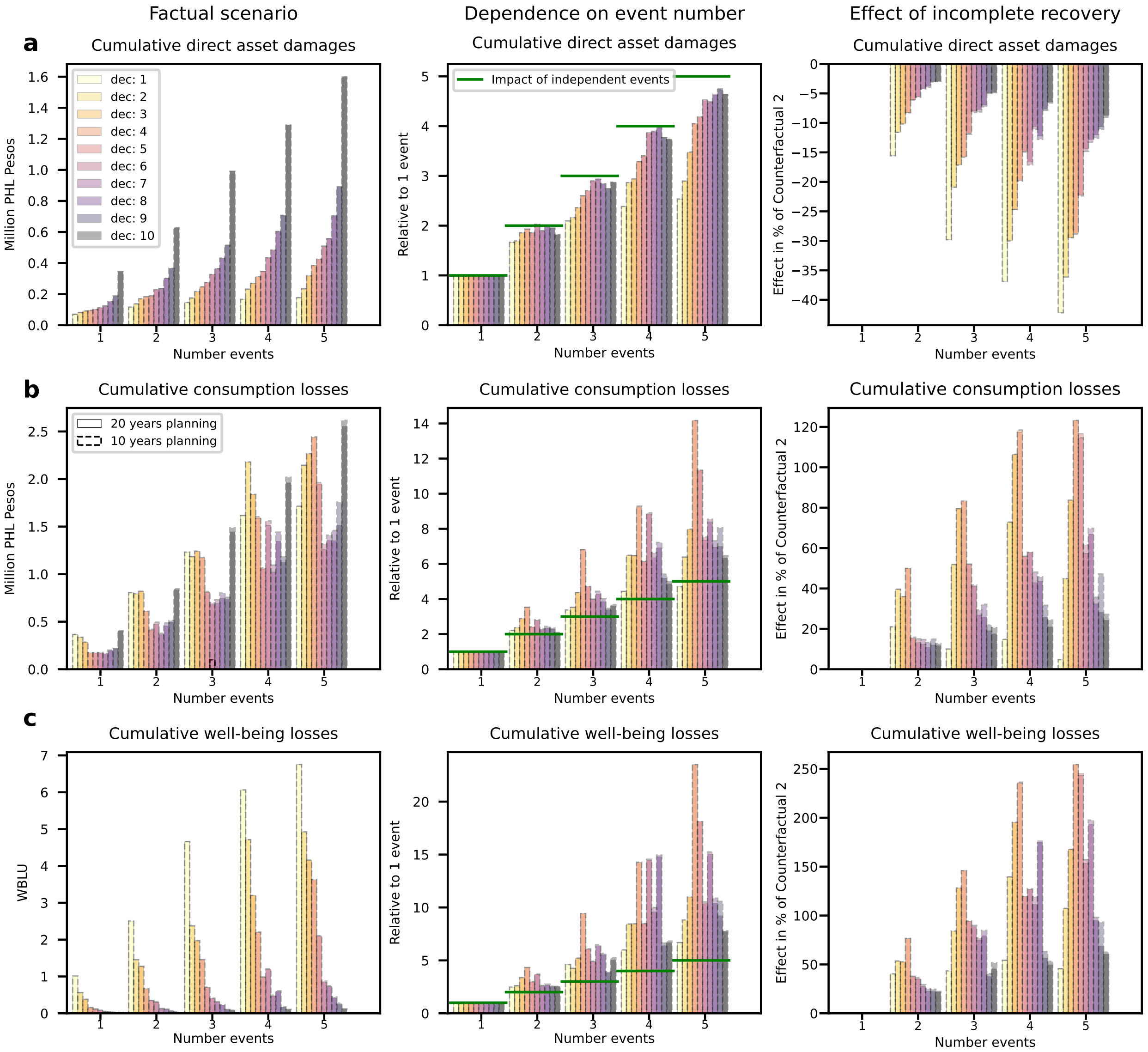

Figure S15. Distributional effects for households in dependence of the number of floods they experience for different planning horizons T_p. Left column: Average cumulative direct asset damages **(panel a)**, average cumulative consumption losses **(panel b)**, and average cumulative well-being losses **(panel c)** for households in each income decile that are affected by 1-5 flood events in the factual scenario. Middle column: Average increase in losses with the number of flood events that households experience relative to the average losses of households that are affected only by one flood event. Horizontal green lines indicate damages and losses that would occur if losses increased linearly with event number. Right column: Relative increase in average losses in the factual scenario where households may not recover between events compared to the counterfactual scenario 2 where full recovery is always possible. Absolute well-being losses are measured in well-being loss units (WBLU) (Methods).

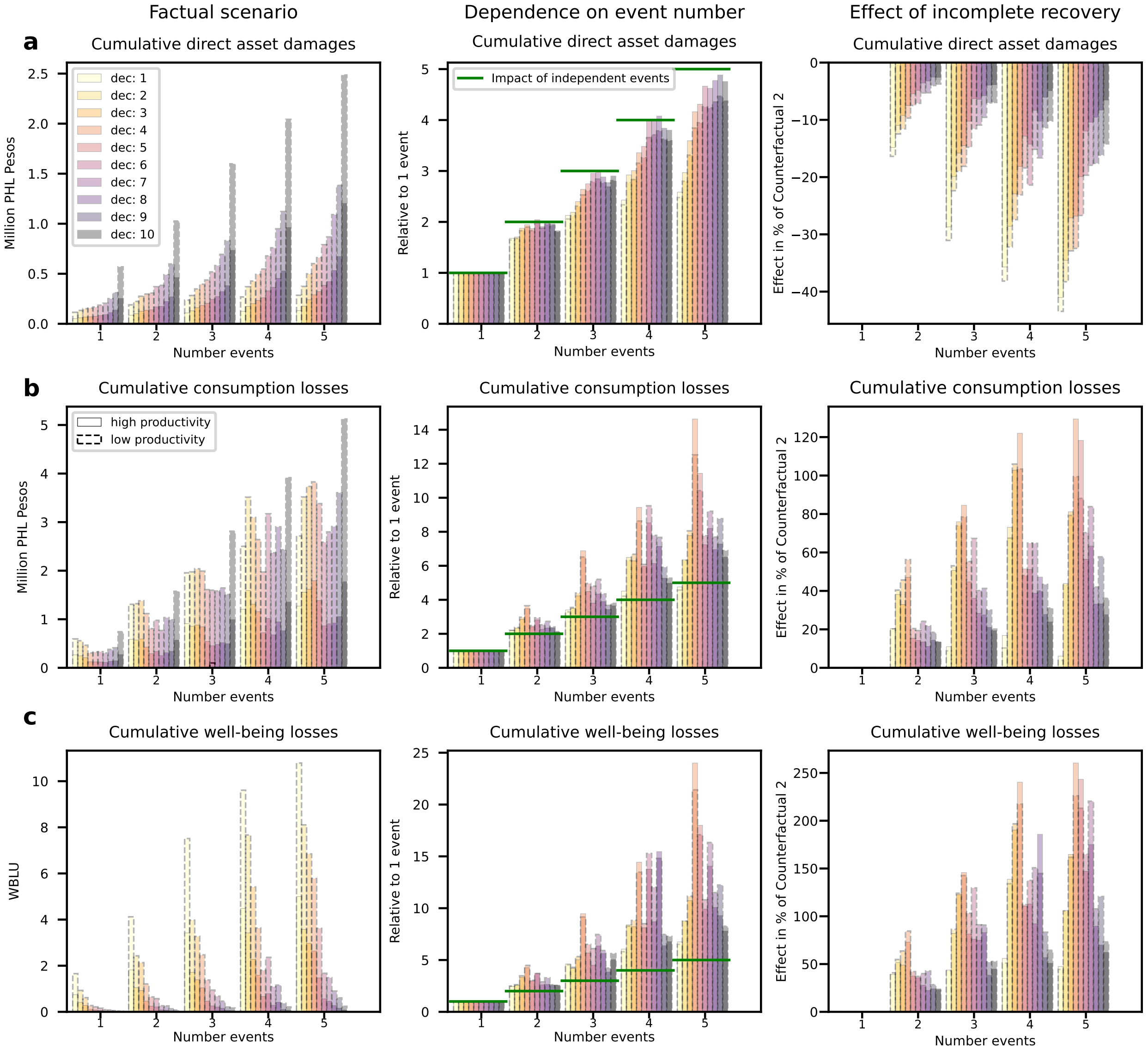

Figure S16. Distributional effects for households in dependence of the number of floods they experience for under varying productivity of capital π. Left column: Average cumulative direct asset damages **(panel a)**, average cumulative consumption losses **(panel b)**, and average cumulative well-being losses **(panel c)** for households in each income decile that are affected by 1-5 flood events in the factual scenario. Middle column: Average increase in losses with the number of flood events that households experience relative to the average losses of households that are affected only by one flood event. Horizontal green lines indicate damages and losses that would occur if losses increased linearly with event number. Right column: Relative increase in average losses in the factual scenario where households may not recover between events compared to the counterfactual scenario 2 where full recovery is always possible. Absolute well-being losses are measured in well-being loss units (WBLU) (Methods).

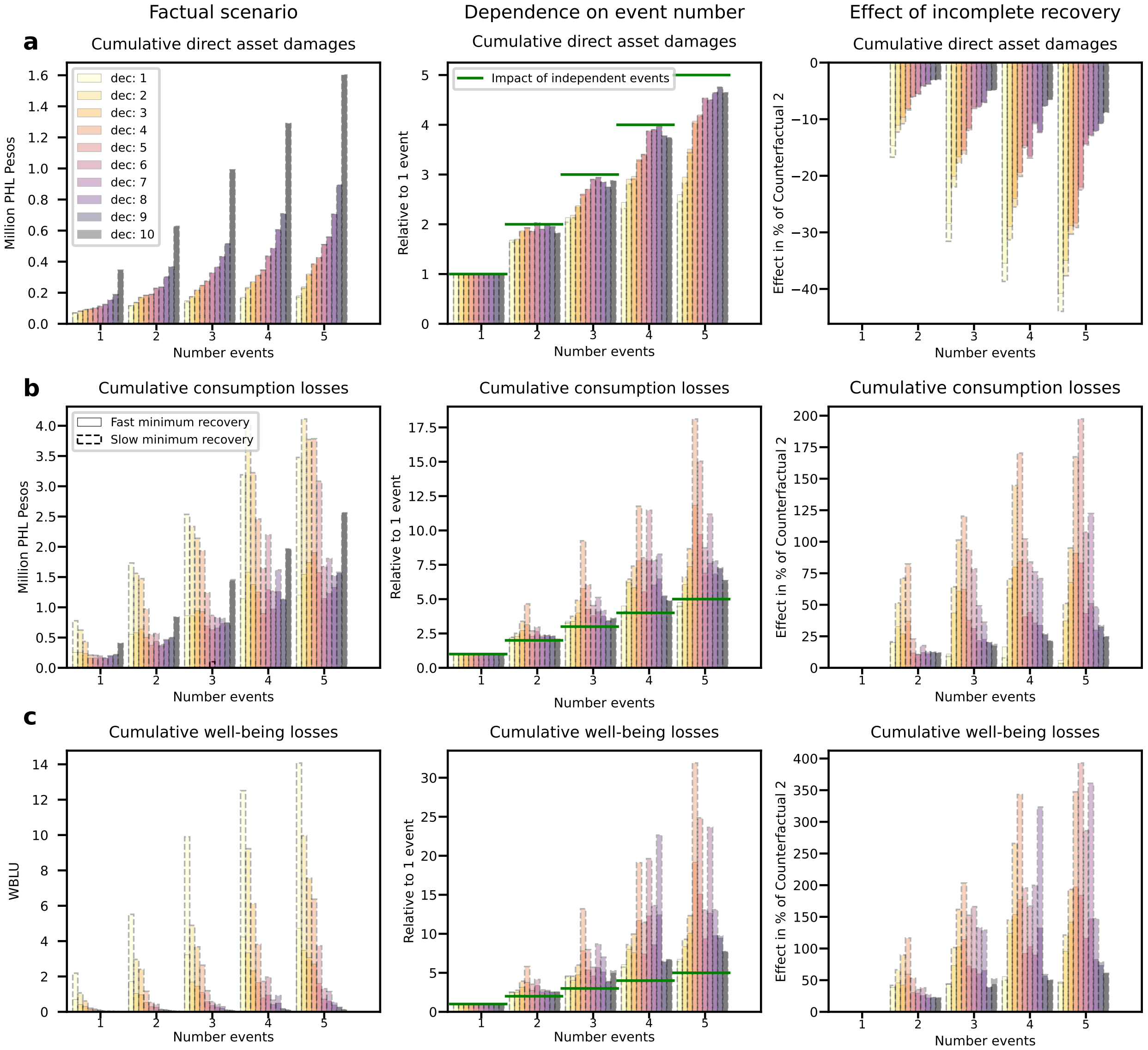

Figure S17. Distributional effects for households in dependence of the number of floods they experience for different minimum recovery rates R_{sub}. Left column: Average cumulative direct asset damages **(panel a)**, average cumulative consumption losses **(panel b)**, and average cumulative well-being losses **(panel c)** for households in each income decile that are affected by 1-5 flood events in the factual scenario. Middle column: Average increase in losses with the number of flood events that households experience relative to the average losses of households that are affected only by one flood event. Horizontal green lines indicate damages and losses that would occur if losses increased linearly with event number. Right column: Relative increase in average losses in the factual scenario where households may not recover between events compared to the counterfactual scenario 2 where full recovery is always possible. Absolute well-being losses are measured in well-being loss units (WBLU) (Methods).

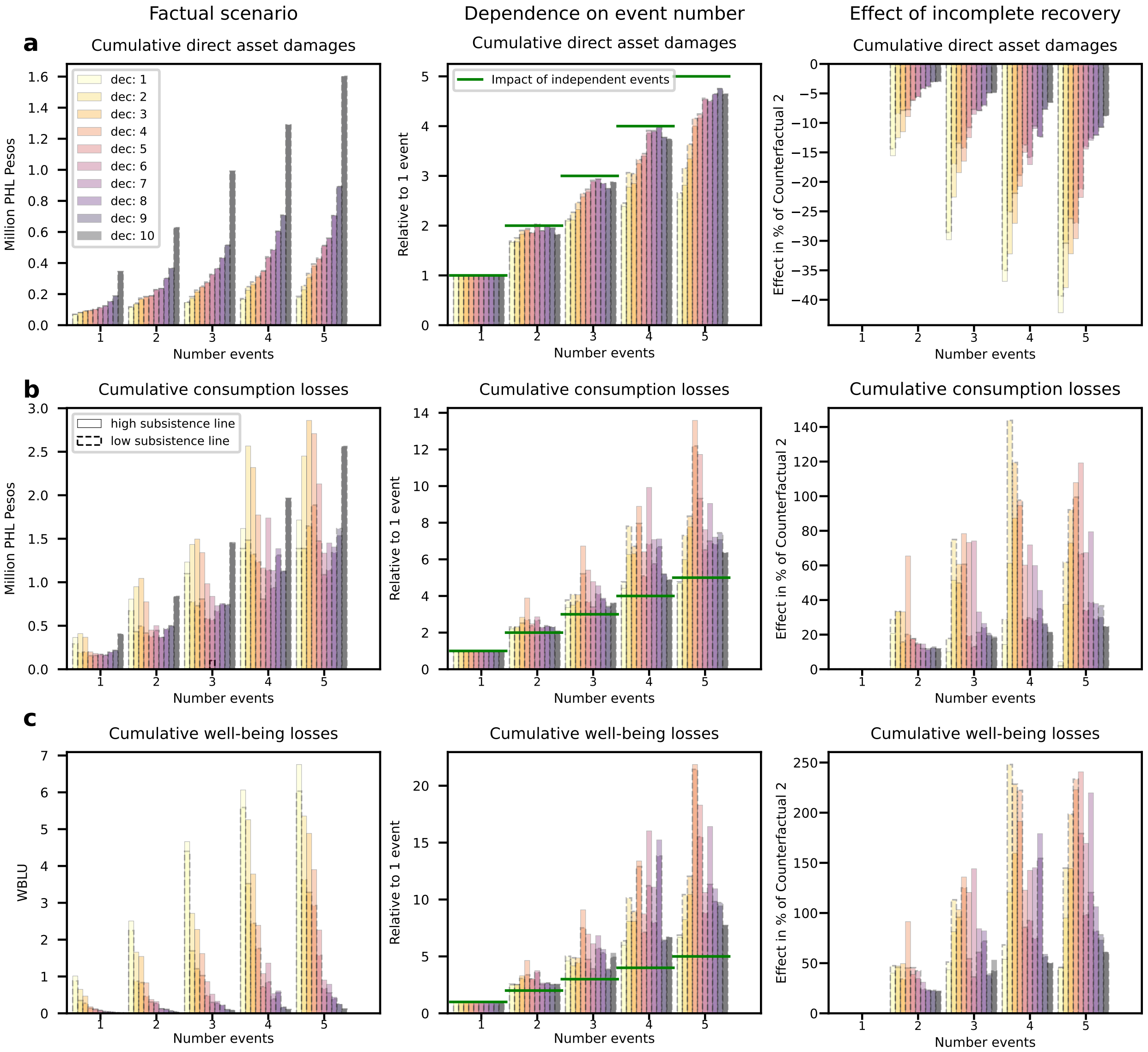

Figure S18. Distributional effects for households in dependence of the number of floods they experience for different subsistence lines. Left column: Average cumulative direct asset damages **(panel a)**, average cumulative consumption losses **(panel b)**, and average cumulative well-being losses **(panel c)** for households in each income decile that are affected by 1-5 flood events in the factual scenario. Middle column: Average increase in losses with the number of flood events that households experience relative to the average losses of households that are affected only by one flood event. Horizontal green lines indicate damages and losses that would occur if losses increased linearly with event number. Right column: Relative increase in average losses in the factual scenario where households may not recover between events compared to the counterfactual scenario 2 where full recovery is always possible. Absolute well-being losses are measured in well-being loss units (WBLU) (Methods).

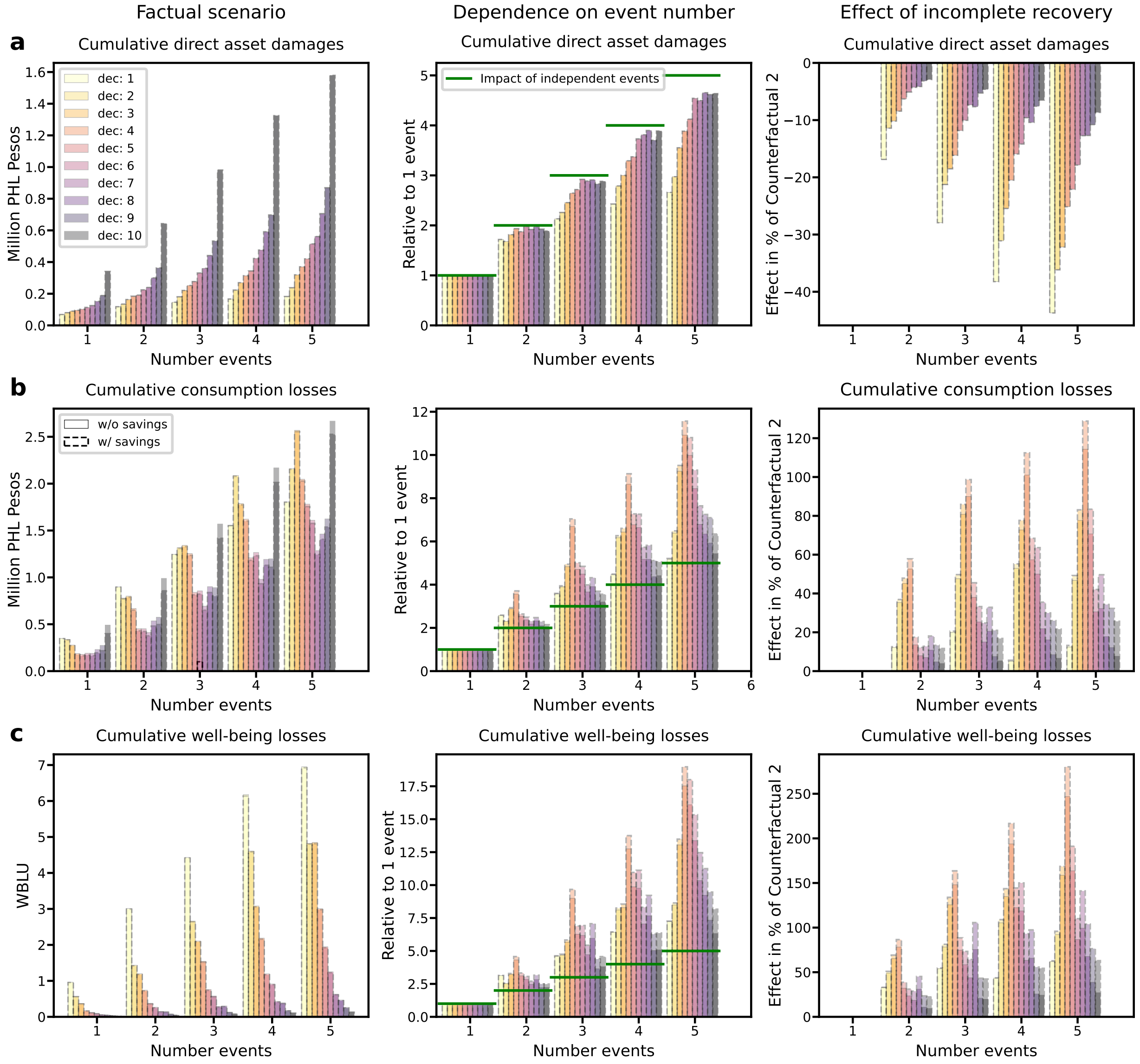

Figure S19. Distributional effects for households in dependence of the number of floods they experience. Comparision of a the "savings" and "no-savings" scenario. Left column: Average cumulative direct asset damages **(panel a)**, average cumulative consumption losses **(panel b)**, and average cumulative well-being losses **(panel c)** for households in each income decile that are affected by 1-5 flood events in the factual scenario. Middle column: Average increase in losses with the number of flood events that households experience relative to the average losses of households that are affected only by one flood event. Horizontal green lines indicate damages and losses that would occur if losses increased linearly with event number. Right column: Relative increase in average losses in the factual scenario where households may not recover between events compared to the counterfactual scenario 2 where full recovery is always possible. Absolute well-being losses are measured in well-being loss units (WBLU) (Methods).

2 Supplementaty Tables

**FIES descriptor**	**Category**	v_h
Strong material (galvanized iron, concrete, aluminium, stone, asbestos	robust	0.14 ± 0.06
Mixed but predominantly strong materials	robust	0.14 ± 0.06
Light material (cogon, nipa, anahaw)	moderate	0.4 ± 0.08
Mixed but predominantly light material	moderate	0.4 ± 0.08
Savaged/makeshift materials	fragile	0.7 ± 0.14
Mixed but predominantly salvaged material	fragile	0.7 ± 0.14
Not applicable	fragile	0.7 ± 0.14

Table S1. Household vulnerability estimated from the FIES. The conversion of building structure to vulnerability factors was adopted from Ref[3]:

Event date	People GPW[4] ∩ GFD[2]	Affected people EM-DAT[5]	$\frac{\text{Affected people}}{\text{GPW} \cap \text{GFD}}$
2002-07-06	529648	25000	0.047201
2002-07-20	99391	7000	0.070429
2003-06-25	80583	10000	0.124095
2003-07-19	89980	20000	0.222273
2003-12-19	159838	97500	0.609993
2004-02-11	220164	20000	0.090841
2004-06-28	24288	1200	0.049407
2004-06-29	88684	0	0.0
2004-07-31	303358	80000	0.263714
2004-08-24	346674	1058849	3.054307
2004-11-19	125839	35000	0.278132
2004-11-23	229551	8000	0.040430
2004-11-29	652651	432000	0.661916
2005-07-27	168604	32782	0.194432
2005-09-15	197874	8000	0.040430
2006-01-26	253292	256641	1.013224
2006-07-11	3327	51680	15.531922
2006-11-30	121877	82915	0.680315
2007-01-10	209753	24508	0.116842
2007-10-27	43850	0	0
2008-02-12	261198	873000	3.342294
2008-05-10	234378	50000	0.213331
2008-07-28	119956	76000	0.633568
2008-12-28	477421	3500	0.007331
2009-01-20	199822	0	0.0
2009-02-02	228882	2000	0.008738
2009-07-25	183902	200000	1.087539
2009-10-02	454278	40000	0.88052
2010-05-29	168077	0	0.0
2011-01-01	173782	10000	0.057543
2011-02-01	60382	2000	0.033122
2011-06-05	308053	15000	0.048693
2011-09-28	33045	180000	5.447144
2011-12-16	317969	1000	0.003145
2012-02-20	375595	25000	0.066561
2012-03-26	9213	0	0.0
2012-12-04	31355	60000	1.913571
2013-01-04	107597	3180	0.029555
2014-06-17	270474	35000	0.129402
2014-09-19	357108	83000	0.232422
2014-12-10	249174	80186	0.321807
2015-10-22	473307	60000	0.126768
2015-12-12	533209	20000	0.037509
2017-12-12	285081	0	0.0

Table S2. Fraction of affected people for selection of affected households. The fraction of affected people for each event describes the ratio of exposed population (intersection of flooded area in the Global Flood Database (GFD)[2] and population[4] and the number of affected people given in EMDAT[5].

References

1. Philippine Statistics Authority. Republic of the Philippines. Results from the 2015 family income and expenditure survey (2015).
2. Tellman, B. *et al.* Satellite imaging reveals increased proportion of population exposed to floods. *Nature* **596**, 80–86, DOI: 10.1038/s41586-021-03695-w (2021).
3. Walsh, B. & Hallegatte, S. Measuring Natural Risks in the Philippines: Socioeconomic Resilience and Wellbeing Losses. *Econ. Disasters Clim. Chang.* **4**, 249–293, DOI: 10.1007/s41885-019-00047-x (2020).
4. Doxsey-Whitfield, E. *et al.* Taking Advantage of the Improved Availability of Census Data: A First Look at the Gridded Population of the World, Version 4. *Pap. Appl. Geogr.* **1**, 226–234, DOI: 10.1080/23754931.2015.1014272 (2015).
5. EM-DAT, CRED. UCLouvain, Brussels, Belgium. https://www.emdat.be/ (2021). Accessed: 2021-09-10.
